# Mental-physical multimorbidity treatment adherence challenges in Brazilian primary care: A qualitative study with patients and their healthcare providers

**DOI:** 10.1371/journal.pone.0251320

**Published:** 2021-05-13

**Authors:** Magdalena Rzewuska, Ana Carolina Guidorizzi Zanetti, Zoë C. Skea, Leonardo Moscovici, Camila Almeida de Oliveira, João Mazzoncini de Azevedo-Marques

**Affiliations:** 1 Health Services Research Unit, University of Aberdeen, Scotland, United Kingdom; 2 Public Health Postgraduate Program, Ribeirão Preto Medical School, University of São Paulo, São Paulo, Brazil; 3 Department of Psychiatric Nursing and Human Sciences, University of São Paulo at Ribeirão Preto College of Nursing, WHO Collaborating Centre for Nursing Research Development, São Paulo, Brazil; 4 Department of Social Medicine, Ribeirão Preto Medical School, University of São Paulo, São Paulo, Brazil; 5 Primary Health Care, Academic Health Services Complex at Ribeirão Preto Medical School of the São Paulo University, XIII Regional Health Department, Unified Health System, São Paulo State, Brazil; Universiteit Gent, BELGIUM

## Abstract

Improved understanding of multimorbidity (MM) treatment adherence in primary health care (PHC) in Brazil is needed to achieve better healthcare and service outcomes. This study explored experiences of healthcare providers (HCP) and primary care patients (PCP) with mental-physical MM treatment adherence. Adults PCP with mental-physical MM and their primary care and community mental health care providers were recruited through maximum variation sampling from nine cities in São Paulo State, Southeast of Brazil. Experiences across quality domains of the Primary Care Assessment Tool-Brazil were explored through semi-structured in-depth interviews with 19 PCP and 62 HCP, conducted between April 2016 and April 2017. Through thematic conent analysis ten meta-themes concerning treatment adherence were developed: 1) variability and accessibility of treatment options available through PHC; 2) importance of coming to terms with a disease for treatment initation; 3) importance of person-centred communication for treatment initiation and maintenance; 4) information sources about received medication; 5) monitoring medication adherence; 6) taking medication unsafely; 7) perceived reasons for medication non-adherence; 8) most challenging health behavior change goals; 9) main motives for initiation or maintenance of treatment; 10) methods deployed to improve treatment adherence. Our analysis has advanced the understanding of complexity inherent to treatment adherence in mental-physical MM and revealed opportunities for improvement and specific solutions to effect adherence in Brazil. Our findings can inform research efforts to transform MM care through optimization.

## Introduction

Multimorbidity (MM), defined as the co-existence of at least two chronic physical (communicable or non-communicable) or mental health conditions in a single individual, is a growing global health concern [1]. Management of these conditions is fundamental to minimize their impact, improve health outcomes, prevent further disability, and reduce healthcare costs [2]. Treatment adherence, referred to as the extent to which a person is able to follow the agreed recommendations from their health care provider (e.g., taking medication, following a diet, and/or executing lifestyle changes), is a key component of chronic condition management [3]. The pharmacomanagement of MM often requires the use of combination of multiple drugs from different classes (i.e., polypharmacy), with a higher number of prescription drugs being associated with non-adherence [4]. Polypharmacy, together with a great deal of associated work required in MM health care (e.g., health monitoring, arranging appointments and enacting lifestyle changes), induce treatment burden–that is the impact on patients’ functioning and wellbeing, imposed by the demands on patient and their caregivers’ time and energy [5]. The burden only increases as the number and complexity of conditions increases [6]; as such co-existing physical and mental health conditions (‘physical-mental MM’) are viewed as ‘a key challenge for healthcare systems’ [7–9]. Non-adherence is a major problem in MM as it can lead to adverse health outcomes, increased healthcare expenditure, and even increased risk of death [10–16]; according to the World Health Organization “increasing adherence may have a greater effect on health than any improvement in specific medical treatments” [3].

Existing literature highlights difficulties in understanding and effecting changes in treatment adherence in MM [17]. This is commonly attributed to management being poorly addressed by current one-disease-at-a-time research evidence and clinical guidance [18]. In response, person-focused integrated care approaches emerged, that stress the need to view MM through a ‘complexity’ lens, with a focus on understanding, as a contextualized whole, parts of multifaceted MM management [18]. This is because ‘health complexity’ is a common feature of MM [7, 19–22], defined as a non-linear and dynamic interaction of physical health, mental health, demographics, social capital; and health and social experience [23], which indeed poses major difficulties for care planning and delivery [24, 25].

MM is common in Brazil’s adult populations, with a prevalence of 24%, highest in older people, women and those less educated [26]. In Brazil, medications are extensively used [27], but the overall prevalence of “low adherence” to drug treatment for chronic diseases is high (30.8%), and even higher in those with multiple chronic diseases or using five drugs or more [28]. Several improvements were implemented in Brazilian primary health care (PHC), where most MM management takes place [29, 30], but its key aspect—treatment adherence—remains vastly under-researched. Only one qualitative study, on medication adherence in hypertensive patients in Brazil [31], was identified by a recent systematic review [32]. Addressing this gap in evidence could inform solutions to common problems in management of MM in Brazilian PHC. The ultimate goal is to inform healthcare providers (HCP) how to effectively coordinate MM care, while incorporating individual patient priorities and preferences in personalized treatment plans for MM patients [33–35].

We report here findings on treatment adherence in a population with mental-physical MM, collected through a large qualitative study, involving in-depth semi-structured interviews with primary care patients (PCP) and HCP in PHC and mental health community settings of the XIII Regional Health Department (RHD-13) of São Paulo State, Southeast of Brazil.

## Methods

### HCP-led research approach

HCP are likely to have unique expertise which can inform different areas of research. Involvement in PHC research can increase service providers’ ability to mobilize resources and enhance clinical practice [36]. Drawing on participatory research approaches, this study was governed, designed, conducted, and interpreted with PHC staff (ACGZ, JMAM, LM, CAO), using their local expertise. The PHC staff is clinically active, involved in HCP education and service management in RHD-13. ACGZ is a registered nurse leading a psychiatric nursing students placement program, with experience in working and teaching in PHC units linked to Ribeirão Preto Medical School, University of São Paulo. LM is a general practitioner and a coordinator of a family medicine residency program. JMAM is a psychiatrist with specialization in PHC, a coordinator of a PHC unit and the training activities in mental health care of that residency program. CAO is an occupational therapist with PHC residency, who importantly contributes to those training activities. The two non-clinical academic authors brought in psychology (MR), implementation science (MR), sociology (ZS) and methodological (ZS) perspectives.

### Study setting

Two distinct main models of PHC coexist in Brazil. A “Basic Health Unit” (“BHU”) is a traditional model with at least one general physician, gynecologist, pediatrician, and nurses and nursing assistants, and optionally community health workers (CHW), responsible for a population of up to 18,000 people [37]. A Brazilian “Family Health Strategy” team (“FHS”) is an newer PHC model, with multidisciplinary professional teams, usually consisting of a least one general practitioner, one practice nurse, one nursing assistant and CHWs (up 750 patients per CHW); responsible for a population of up to 3500 people [29, 37]. FHS focuses on the use of CHWs, who are often members of the communities in which they work, so therefore have valuable understanding and relationships with patients. Compared to families not registered with FHS or with private healthcare plans, adult FHS enrollees were found to be more satisfied with received care, in particular urban dwellers, women, and the very poorest [38]. FHS coverage has been linked to enhanced aspects of care, including a decreased rate of hospitalizations for certain ambulatory care-sensitive chronic diseases [39]. Overall, 53.4% of the households in Brazil were registered with FHS in 2013 [40].

São Paulo state is administratively divided into seventeen RHDs (health boards). RHD-13 consists of 26 municipalities [41], with a total of 1,327,989 inhabitants in 2010 and Ribeirão Preto its biggest city [42, 43]. Participants were recruited from nine cities in RHD-13, where the HCP who led this project are clinically and academically active. PHC units with BHU or FHS teams only and the two mixed [30] were targeted to capture variability of views and experiences across these two main types of PHC units. To gain multiple perspectives and validate data [44] we aimed to explore views of both HCP working in those units, as well as PHC atendees. For data validation purposes, we also included community mental health service managers and specialists supporting these units.

### Sampling

We used a purposive maximum variation sampling strategy that offered the best opportunity to reach or at least closely approach data saturation (i.e., no ‘new’ themes identified and theoretical insights gained [45]). We focused on variation across participant groups (PCP and four HCP groups (nurses, PHC unit managers/not managers, physicians, mental health professionals, community health workers/nursing assistants)), then type of healthcare unit (FHS, BHU and community mental health service) and municipality size. There is no set rule for determining sample size in qualitative studies [46], but it has been suggested that 12–20 participants would be enough, when looking to achieve maximum variation sampling [47]. Therefore, with five participant sub-groups, we set out to interview around 80 people (an average of 60–100) but were flexible to continue or stop recruiting, depending on whether new interviews were still adding to our understanding of identified themes.

### Eligibility

Adults (aged 18 years or older), PHC attendees, with mental-physical MM were eligible for inclusion. Mental-physical MM was defined as having at least one recognised physical chronic non-communicable disease/conditions (i.e., diabetes, hypertension, heart disorder and/or arthritis) and co-existing mental disorders (i.e., depression and/or anxiety). Knowing that patients would be identified from paper-based health records, we selected conditions/disease from the two most frequent clusters of MM in Brazil [26] that we were certain PHC teams commonly manage and will be able to identify from their health records. That said, the included PCP were encouraged to talk about a condition(s) they felt was important and relevant. Based on physicians’ knowledge about their patients, we excluded PCP presenting with a serious illness or cognitive impairment.

Eligible primary healthcare HCP were physicians, nurses, nursing assistants and CHWs as well as psychologists; and community mental health services managers, all with minimum 6 months experience in their current role.

### Participant recruitment

Health care service units were approached between November 2015 and March 2016. The terms of access were agreed a priori with service managers. The research team directly approached HCP by email or phone. Potential patient participants were identified and approached by PHC staff, who made them aware of the opportunity to give an interview by handing out and for illiterate patients also reading out loud, a short flyer and a formal letter of invitation.

Participation was voluntary. Participants received written information at least 7 days before an interview. On the day of the interview, an interviewer obtained written or verbal informed consent, collected demographic information, and made field notes. Participants were free to withdraw at any time and without giving a reason.

### Data collection

This study used one-off semi-structured in-depth interviews. Our intentions were to collect both ‘thick’ (a large sample size) and ‘rich’ (many layered, intricate, detailed, nuanced) data that covered all key aspects of the PHC process; and incrementally report them. Questions were informed by the PCP and HCP versions of the Primary Care Assessment Tool-Brazil, based on the theoretical model of PHC attributes developed by Starfield—first-contact accessibility and use; continuity; comprehensiveness and coordination; family centredness, community orientation and cultural competence [48–50]. Following piloting with HCP and PCP, HCP topic guides tailored to specifc roles of different professional were developed, and also a patient topic guide—adapted to the needs of people with very low eductional levels (a factor linked to both PHC use and MM [26] in Brazil), including techniques aimed to overcome communication challenges, inarticulateness, frame of reference and the concept of time [51]. Topic guides included lists of open-ended questions and prompts (see S1 Appendix). Participants were prompted to detail process and reflect on factors that influence their and others decision to engage or disengage with a specific behavior.

Interviews, according to preference, were performed at PHC units or at premises of municipal health secretariats, and in the case of patients, also at their homes. The interviewers–all trained and experienced—included four women and one man, of which two were post-graduate HCP, two post-doctoral researchers and one post-doctoral HCP. Amongst the interviewers were two authors (MR, JMAM). Interviewers had no relationship with interviewees prior to study commencement and they introduced themselves as researchers interested in understanding aspects of healthcare delivery process for PHC patients with mental-physical MM. Interviews were conducted between April 2016 and April 2017.

### Participants

Three HCPs chose not to participate (one physician, one nurse, one nursing assistant) due to lack of time, and two patients for unknown reasons. We interviewed 81 participants (62 HCP and 19 PCP). The average interview length with HCP and PCP was 94 minutes (range: 48 to 130 minutes) and 75 minutes (43 to 126 minutes), respectively. Demographic characteristics of PCP and HCP are shown in Tables 1 and 2 respectively. Overall, participating PCP were mostly older women, FHS enrollers, small cities dwellers, with no education or incomplete fundamental education, with three or more mental-physical comorbidities (used for study inclusion). HCP were mostly physicians, registered nurses acting as PHC managers, and CHW, working in units with FHS teams, with an average 7 (CHW) to 11 (physicans) years of experience as HCP.

**Table 1:** Characteristic of interviewed primary care patients (PCP) (N = 19).

Characteristic type	n
**Age category (years)**	
18–30	1
31–40	1
41–50	2
51–60	5
60–70	9
>70	1
**City size**	
10,000<	0
10,001–20,000	4
20,001–30,000	3
30,001–40,000	1
40,001–60,000	2
60,001–80,000	2
120,000–599,000	0
≥600,000	7
**Gender**	
Female	16
Male	3
**Type of PHC unit**	
Mixed FHS-BHU	3
BHU only	3
FHS only	10
FHS with TSSP	1
University FHS	2
**Educational level**	
No education/ complete fundamental	10
Complete fundamental/ incomplete intermediate	4
Complete intermediate/ incomplete superior	4
Complete superior	1
**Multimorbidity pattern**^**a**^	
DM-CHD-AR-A-D	2
DM-HT-D	4
DM-HT-A	2
DM-HT-A-D	3
DM-HT-CHD-A	4
HT-AR-D	1
HT-A-D	2
HT-D	1

A, anxiety; AR, arthritis; BHU, Basic Health Unit; CHD, coronary heart disease; D, depression; DM, diabetes mellitus; FHS, Family Health Strategy; HT, hypertension; TSSP, team of specialists supporting a primary health care unit.

^a^ We present patterns of conditions used as eligibility criteria only.

**Table 2:** Characteristic of interviewed health care professionals (HCP) (N = 62).

Characteristic type	Community mental health services managers	PHC community health workers	PHC	PHC	PHC physicians	PHC	PHC
						registered nurses–not managers	registered nurses -
			mental health professionals	nursing assistants			
					(n = 12)		
				(n = 8)			unit managers
		(n = 13)	(n = 5)				
						(n = 5)	(n = 13)
	(n = 6)						
**Age category (years)**							
18–30	0	2	1	1	4	1	2
31–40	2	4	2	4	6	1	7
41–50	1	4	1	2	1	0	2
51–60	3	2	1	1	1	3	1
60–70	0	1	0	0	0	0	1
**City size**							
10,000<	0	1	0	1	1	0	1
10,001–20,000	1	2	1	0	1	0	2
20,001–30,000	0	1	0	1	1	0	2
30,001–40,000	1	1	1	1	2	0	1
40,001–60,000	1	1	0	0	0	0	1
60,001–80,000	1	1	1	1	1	0	2
120,000–599,000	0	2	0	1	1	0	2
≥600,000	2	4	2	3	5	5	2
**Women**	5	11	5	7	4	5	11
**Type of PHC unit**^**a**^							
Mixed FHS-BHU	NA	1	1	2	2	0	1
BHU only	NA	0	0	2	3	1	4
FHS only	NA	9	2	4	5	2	7
FHS with TSSP	NA	2	2	0	2	0	1
University FHS	NA	1	0	0	0	2	0
**Years of work in health care (average)**	20.4	7.3	7.2	12.5	11.0	21.6	13.8

BHU, Basic Health Unit; FHS, Family Health Strategy; NA, not applicable; PHC, primary health care; TSSP, team of specialists supporting PHC units.

^a^ Community mental health services managers worked in: mental health outpatient services (MHOS) (n = 3) and mental health community centres (MHCC) (n = 3)

### Analysis

Interviews were audio-recorded and transcribed verbatim [52]. All transcripts were checked for accuracy but not returned to participants for comments, given the advantages of interviewee transcript review are small and the added time and effort is substantial [53]. Data was analysed in Portuguese to preserve the original meanings, using thematic content analysis. Transcripts were formally coded through inductive coding for common phrases that discussed the same idea or had the same meaning, incorporating principles of constant comparison [54]. We started open coding by reading the first ten available interview transcripts word by word and line by line. After completion of open coding we determined the preliminary codes that emerged from the transcripts and then coded the remaining transcripts with those codes. When encountering data that did not fit any of the existing codes, we added new codes. Data coding was conducted using the Atlas ti. Software (Scientific Software Development GmbH, Berlin). Next, using coding reports we grouped similar codes into categories, reorganized them into higher order categories [55], then grouped, revised, and refined ensuring the categories were mutually exclusive [54]. The contents of the categories were then compared between participant sub-groups and types of PHC units. Finally we identified emergent and recurring themes within and across categories, organized them into sub-themes and themes, then compared them against each other and finalized [56]. To ensure consistency and reliability of the coding and themes generated, three researchers (ACGZ, MR, JMAM) jointly conducted this process. For this article, our multidisciplinary team of healthcare service researchers (MR, ZS) and academic primary care staff (ACGZ, LM, CAO, JMAM) jointly selected themes and sub-themes associated with ‘treatment adherence’ and linked them into meta-themes. Indicative quotes representing each theme/sub-theme were chosen through a discussion and consensus between the team members.

To ensure trustworthiness and validity of this study, data obtained from PCP interviews and HCP interviews was triangulated [44]. We kept memos (reflective notes), which we considered both during the data analysis and when writing the discussion section. Briefings and analytic triangulation with peers with expertise in qualitive research were held throughout the study.

### Ethics approval

The project was approved by the Research Ethics Committee of the Clinics Hospital of Ribeirão Preto Medical School, the Board of Health Managers of RHD-13 and the Secretariat of Health of the Ribeirão Preto city. CAAE No: 48183515.9.0000.5440.

## Results

Identified themes associated with treatment adherence were grouped into ten meta-themes, discussed below. The thematic tree can be found in S2 Appendix. Each theme and sub-theme in a meta-theme was assigned a number (stated in round brackets), for which relevant representative exemplary quotes, anonymized and translated to English, were selected; some of which can be found provided below, and a full list of quotes can also be found in S2 Appendix.

### 1) Variability and accessibility of treatment options available through PHC

To begin with, marked disparities in treatment access emerged from interviewees’ descriptions; that is available treatments’ options varied from one service to another with a much wider range of treatment modalities being reported by HCP working in FHS or mixed FHS-BHU units, compared to those in BHU only units. (1.1) ‘Usual care’ includes prescriptions, a selected list of medicines subsidized by the government, scheduled follow-ups, health education, home visits and referrals to secondary mental and physical health services and social services. Interviewees reported some units offering advanced multifaceted approaches, including PHC practice-based group activities (e.g., physical exercise, crafts, healthy eating); complementary therapies; multidisciplinary meetings regarding cases and/or specific themes; and specialists supporting PHC teams. (1.2) PCP expressed strong preference for multidisciplinary and even integrated PHC-based care, including appointments with specialist physicians, mental health, and allied health professionals. PHC staff also univocally stressed the value and preference for collaborative multidisciplinary approaches. As such, strategies to expand and integrate care delivered by specialists with the care delivered by PHC doctors and nurses in that setting (e.g., actions delivered by physical educators and nutritionists of the “*HIPERDIA*” program for patients with hypertension and diabetes) were viewed as positive and necessary in terms of mitigating current limited access to specialised care. (1.3) In addition to treatment options received via PHC, PCP widely discussed also using their own coping strategies to manage psychological aspects of their physical and/or mental conditions, including praying, diverting attention from a disease (by reading, playing games and craft), crying and talking with relatives, neighbours and friends about everyday life.

*(1*.*1)* ‘‘*I think that the treatment [for chronic diseases] tend to be*, *here* *at a Basic Health Unit*, *more of medication and guidance*. *We don’t have more to offer*, *this is usual*.*” (physician (PHY) 9 –BHU*, *female*)

‘‘*We have here [at the unit] a social worker*, *family doctor*, *speech therapist*, *psychologist*, *nutritionist*, *who is currently on maternity leave*. *Then*, *we have the unit’s physiotherapist too*, *okay*. *I think it’s just (*..*) The community workers (*..*) That is*. *There is a practice nurse and an assistant*, *right*? *[*…*] there’s the nursing assistant*. *There is auriculotherapy too*.*” (CHW 8 –FHS with team of specialists supporting a PHC unit (TSSP)*, *male*)

*(1*.*2)* ‘‘*[We could offer better mental care] if we had access to a TSSP*, *which has a psychologist*, *which provides access to psychology*.*” (registered nurse–a PHC service manager (RN-M 1)–FHS*, *female*)

(*1*.*3)* “*Yeah*, *I make my rugs*, *sometimes I play a game*, *sometimes I’m alone and I play a game you know*? *Then I am distracted [*…*] My granddaughter has a cell phone and has a lot of games they have puzzles*, *lots of things there (*..*) as long as I am distracted [it’s ok]” (PCP 15 –BHU*, *female*)

### 2) Importance of coming to terms with a disease for treatment initiation

(2.1) Some PCP described how validation from a family member/or other significant other helped them out of that state of denial/indifference. HCP reflected on the underlying process of coming to terms with illness and accepting treatment, as fundamental to treatment initiation. In their experiences, some PCP respond to illness with denial, which they considered detrimental to treatment initiation. (2.2) HCP observed that PCP struggle to transition to acceptance of illness and its management, without having ‘experienced symptoms’ to move them out of denial. This was felt especially problematic for the management of asymptomatic diabetes and hypertension. This resonated with the experiences of some of the interviewed patients. As such, having physical symptoms was viewed by HCP as more likely to trigger the process of adjusting to illness. Disease complications, such as cardiac arrest, were felt to act as facilitators for PCP coming to terms with their disorder and committing to treatment.

(2.1) “*I was shaken out [of my apathy] by my husband*, *he said*: *“It is not like that*, *you cannot do this*, *you cannot throw yourself on a bed because you have diabetes*, *diabetes will not kill you*, *what will kill you is your dismotivation*, *because you are too discouraged*, *it’s not like that”*. *He said*: *"Go out and talk to the doctor*, *she will explain what you have to do"*. *That’s where I went at that time and started to treat*.*” (PCP 2 –FHS*, *female*)

(2.2*)* “*One day*, *I was very ill*, *and the doctor was the first thing he said*: *“has anyone done a blood test to see if this girl does not have diabetes*? *[*…*] Before you put the serum in [saline solution]*, *said*, *let’s do it*.*”*. *It was almost four hundred (*..*) But I didn’t believe it*. *I didn’t believe it because I didn’t feel anything*, *I didn’t feel much (*..*) lots of urine and [drinking] water*, *all that*. *[…] It reached a limit*, *four hundred and so much [blood glucose]*. *Then I stayed in the ICU*, *monitored*, *everything*. *It was a horrible experience (*..*) I said to myself*: *“I’m dying” (*..*) So*, *as you are a layman on the subject*, *you say*: *“man*, *which one is going on*, *why am I here*? *What did I do that was so wrong*? *[…] [We] start to understand that you really have a serious health problem*, *so that you have to take action*. *And this is what I took (*..*) I became aware*. *So I started the treatment*.*" (PCP 17 –FHS*, *female*)

### 3) Importance of person-centred communication for treatment initiation and maintenance

Throughout their interviews most PCP and some HCP (mostly physicians) stressed the importance of person-centred communication for treatment initiation and maintenance. (3.1) They extensively discussed the value of personalised rich PCP-HCP interactions that involved demonstrating interest in a person, a commitment to including the perspective of the person living with disease, and an understanding of who the person is, their life history and preferences, that helped to open up conversations and foster trusting relationships. Person-first, strengths-based, empowering language, was felt to enhance patient motivation. Furthermore, PCP stressed that a depersonalised, mannerless, disrespectful, and rushed communication style that some of them experienced hindered or even impaired their interest in treatment compliance. (3.2) While comprehensive and understandable information about treatment options were considered essential to effective communication by both PCP and HCP; that said, in practice this quality of communication was experienced variably across patients and specific HCP. Patients found strategies, such as examples of good and bad dietary choices, types of physical activity, written individualised information or demonstrations (e.g., application of insulin) helpful. Time was viewed as an essential enabler of communication–for example, patients felt that when possible longer consultations (20–30 mins) were needed to discuss symptoms, treatment options and address doubts. On the other hand, the PCP considered ‘scare-tactics only’ approaches unhelpful, as well as brief consultations with small and very general information.

*(3*.*1)* “*Having this [kind of] doctor to attend you*, *right*? *What else do you want*? *In addition to creating a bond with him [*…*] it becomes so*, *trustworthy*. *You come here and make effort*, *and he’s there*. *Then he would say*: *“Look*, *so-and-so*, *how are you*?*” I mean*, *he knows your name*, *right*? *He knows who you are*. *[He asks*:*] "Is everything* *ok*? *Mom*, *husband”*, *and so on*.*” (PCP 17 –FHS*, *female*)

“*I think for the patient to adhere to the treatment that you are proposing to him/her*, *he/she has to trust what you are saying*. *So*, *listening to the patient is very important in this case because he/she will realise that you are interested in what he/she has*, *you are not going to just give a medicine*, *you are interested in helping him in this other part*. *It helps a lot in patient’s adherence to treatment*, *him/her accepting your suggestions in relation to this [what he has]*.*” (PHY 6 –mixed FHS & BHU*, *male*)

*(3*.*2)* "*[A] piece of paper*. *In writing [*…*] he [a doctor] explained that I had to change the diet a lot*. *Eat more cooked things*, *less fried food*, *certain types of fruit*, *do not eat much*, *eat in between*. *Oh*, *and not eating too much greasy stuff [*…*] And also not using alcohol*, *which I never did anyway*, *I never drunk*. *So I think this helped a little too*.*” (PCP 13 –mixed FHS & BHU*, *male*)

*’*’*So*, *we try to make drawings*, *use every possible resource and available strategy*, *to try to make the patient understand what he/she has*, *because if he/she doesn’t understand*, *he/she won’t adhere*, *if he/she doesn’t understand what each medicine is for*, *why he/she has to use that*, *why he/she cannot eat sugar*, *he will not treat it*. *’’ (registered nurse (RN) 5 –university FHS*, *female*)

### 4) Information sources about received medication

HCP extensively discussed the challenges of establishing what treatment a patient had received. (4.1) Physicians unequivocally felt that electronic health record (EHR) systems were incomplete (fragmented, limited to municipal public health system and medication), paper-based health records were poorly completed (e.g., physicians fail to record prescriptions) and counter-references were rarely sent to the PHC services, which acted as major barriers to knowing what treatment a patient received. They reported routinely reviewing patients’ health records or optionally pharmacy computer records in an attempt to track prescribed medication. (4.2) To complete missing information on medication, HCP reported relying on patients reports, who often struggle to remember their medications, which some BHU physicians admitted proved difficult for them due to time restrictions and doctor turnover. In the context of PHC units where physicians frequently change, PCP expressed however their frustrations with having to repeatedly report their complex healthcare history. (4.3) FHS team members and PCP reported missing information being also gathered by asking a patient to present prescriptions or medication to physicians during consultations (4.4) or to CHW during home visits.

*(4*.*1)* ‘‘*And then I find out*, *asking*, *most of the time or in the same way*, *I will even check in Hygia [a EHR system]*. *I go to ‘medications received’ [at a mental health community centre (MHCC)] and I see*, *but it depends a lot on the person’s information*. *It happens not having a letter of reference*, *nor access to the electronic health records*.*” (PHY 10 –FHS*, *male*)

*(4*.*2)* “*I usually find out through the patient*, *by asking the patient*: *“Did you go there [another healthcare service]*? *What has been done [there]*? *Did you get better*? *You didn’t get better*?*” Through the patient*, *it’s not through the doctor here*, *in the system*.*” (PHY 6 –mixed FHS & BHU*, *male*)

*(4*.*3)* “I *usually ask the patient to bring the prescription*. *There are patients who have a difficulty*, *that is*, *explaining what they are taking*, *you know*. *So*, *I can’t understand through the conversation*. *I say "so*, *when you come to therapy*, *I would like you to bring your prescription"*. *Don’t have the recipe*? *Bring the medicine box*, *of everyone you have taking*. *Then I can do it*.*” (primary care mental health professional (PCMHP) 3 –FHS*, *female*)

*(4*.*4) ‘*‘*Usually you get there [to a patient’s house]*, *they come and sometimes they even come with the little bag*, *you know*, *and then*, *they already show you the medication they take*, *there are usually all these medications they take*: *“I took this*, *took this*.*” (CHW 7 –FHS*, *female*)

### 5) Monitoring medication adherence

HCP described the burden of establishing medication adherence. (5.1) In FHS units, routine monitoring of disease (e.g., glucose, blood pressure) serves as a primary proxy for appropriate medication use. (5.2) Through their trust and understanding with patients, FHS physicians also discussed using subtle signs of poor medication adherence, such as a patient having difficulties with providing descriptions of drug regimes, patient-reported accumulation of medication or unexpected patterns of patients’ requests for prescription renewal. Some PCP described accumulating medication as a precautionary measure for emergency situations, which gave them a sense of safety. (5.3) PHC pharmacy computer records were often used and viewed by physicians and nurses as the most reliable source of medication adherence, where it can be seen what has been prescribed, and if a patient either accepted or intentionally refused medication. The stricter surveillance rules regarding psychotropic medications was felt to facilitate the process of monitoring uptake through pharmacy records. (5.4) FHS team members also mentioned being informed about non-adherence by a patient’s family members. (5.5) HCP described domestic visits of CHWs and nurse technicians as critical to detect and confirm suspected non-adherence, which visits PCP extiensively valued.

*(5*.*1*.*)* “*I usually find out because they come to the appointment with uncontrolled pressure or diabetes*, *the medical test results are very bad*. *[*…*] Sometimes*, *like I said*, *we only know because his/her pressure is out of control*, *his/her exams are so bad or he/she is not feeling well*, *he/she has some symptoms of the disease*.*” (PHY 6 –mixed FHS & BHU*, *male*)

*(5*.*2)* "*And*, *sometimes*, *when you prescribe medicine for the patient*, *he/she says*: *" Ah*, *this one you don’t have to [prescribe] because I have enough at home"*. *Have at home*? *Something is wrong*. *Then I say*: *“Even though you have it*, *it will come out on prescription and you should take it the right way”*. *So*, *like this*, *it is to take them every day*, *it is not to accumulate them*, *I make it very clear*. *This business of leaving a lot at home is not the [right] thing*. *The pharmacy delivers for a maximum of two months*.*” (PHY 8 –FHS*, *male*)

*(5*.*3*.*)*“*The pharmacy [staff] always makes a note on the back of prescription the day it was taken and has the date of the prescription*, *I put the date on all of them*, *so if it was in August in January*, *it has to be taken again*, *January is in May again*, *I am doing it now it starts in September again*. *So*, *if he comes back after that*, *we ask him what happened*. *[*…*] Psychotropic medications are better supervised and that helps us too” (PHY 5 –FHS with TSSP*, *female*).

*(5*.*4)*"*I call a spouse–“ah*, *for the next appointment*, *could you bring your accompanying person*?*” and try to talk to the spouse*, *[asking] if the patient is adhering*, *if is using medication*, *if is behaving*. *So there are ways for us to try to find out if this is happening*.*” (PHY 9 –BHU*, *female*)

*(5*.*5)* “*She [a CHW] always comes here*, *because she books my appointments and if she doesn’t come*, *she doesn’t let you down*, *the phone rings*, *she sends my neighbour*. *My head is forgetful*, *but the girls [CHWs] come here*, *they say*: *“Mrs [name]*, *come [to PHC unit] the day” (PCP 1—FHS*, *female*)

“*I had a patient for whom we used to separate medication*, *so that she could take it right*. *Basically*, *she was hidding the pills*, *then once we went to visit and I was looking at the medication she takes*, *then she opened a little bottle*, *it was full of the medication that we had separated for her*, *so she didn’t take the medication even though we separated it by day*, *by schedule*, *taking the guidelines*, *she didn’t want it*.*” (CHW 5 –FHS*, *female*)

### 6) Taking medication unsafely

HCP, mostly FHS team members, discussed the widespread problem of unsafe use of prescribed medication, that caused them much frustration, echoing PCP’ accounts of widely experienced difficulties with taking medication as prescribed. (6.1) Amongst described problems were issues with taking medicine on time; (6.2) using the right amount (skipping doses or taking half doses of a prescription drug (on purpose or forgetting)); (6.3) checking with HCP before stopping (not taking prescription medicine until it is finished or until a doctor says it is all right to stop); and (6.4) reporting problems (PCP not informing a, HCP not taking a note of side effects, or concerns about safety). (6.5) Many physicians also believed that patients share medication (e.g., take medicines prescribed for another person and give to someone else), but no description was encountered in PCP’ descriptions that would explicitly confirm this.

*(6*.*1)* "*So*, *for example*, *this [medication] is to be taken after breakfast*, *[so patient would say] " when I don’t have breakfast*, *I don’t take medication"*. *It happens sometimes*. *Then*, *"it’s for taking after lunch*, *I didn’t have lunch*, *so I didn’t take it"*. *Then I say*, *“even if you don’t have lunch*, *take it at the time it would be for lunch” because [a patient] confuses things*, *he/she thinks it’s only with breakfast*, *only at lunch*, *just at dinner” (PHY 5 –FHS with TSSP*, *female*)

*(6*.*2*.*)* “*It is so much medicine these days that I spent three days without taking my heart [medication]*, *in the room I was fainting*, *I was very tired*, *very short of breath*, *then I realized—my medicine bag*. *I realized that I wasn’t taking it*, *my daughter even got mad*: *“Mother for the love of God*, *the most important thing”*. *She went running*, *bought it*, *I started taking it*, *it’s to take two per day*.*” (PCP 1—FHS*, *female)*

*(6*.*3)*“*That I’m taking medicine*, *these medicines that you take for depression are addictive*. *So I start taking the medicine*, *[as soon as] I start to feel good*, *I stop*. *[*…*] they [antidepressants] to give you that thing*, *to give you that up in life*, *they throw you all the way down*. *Then you feel more depressed*, *you feel like that*, *a total wreck*.*” (PCP 8 –mixed FHS & BHU*, *female)*

’’*[A] lot of people think that hypertension [involves] taking medication whenever they want*, *when they want*, *you know; they stop*, *discontinue the treatment’’ (RN-M 13 –BHU*, *female)*

*(6*.*4)*“*Then I said [to a patient]*: *“Ah*, *very well*. *Then you felt you should stop taking what was prescribed for blood pressure because it is giving you*, *you think that the pressure medication is giving you dizziness*? *When do you get a follow-up appointment*?*”*.*”Ah*, *it’s next month" [patient responded]*. *I said*: *“So*, *you are going to do the following*: *you go to the [primary care] unit*, *at seven in the morning*, *you go to talk to the nurse*. *If it is the case*, *the nurse who will decide to show you to the clinician*, *the clinician [*…*] Tell the clinician what is happening to see if it really is the pressure medicine*, *if it is the dizziness medication*, *if it is the thyroid one*, *what’s going on*.*” (CHW 11 –FHS*, *female)*

*(6*.*5)* “*I even suspect that they create a black market for medications*: *they simulate situations*, *simulate symptoms in order to obtain*, *for example*, *medications and be giving to each other*. *I have exposed this [type of] situation a lot*. *People end up lacking medications*, *I see that it ends sooner [than expected]*.*" (PHY 3 –FHS*, *male)*

### 7) Perceived reasons for medication non-adherence

Broadly speaking, PCP and FHS team members exclusively shared their views on reasons for medication non-adherence. (7.1) They felt that access to prescription drugs had a major impact on discontinuation of medication, including difficulties with access to medication, failure to renew prescriptions on time, losing prescriptions, prescription expiry, shortage of medication supply via policy of pharmaceutical assistance programs, and patients lacking financial resources to purchase medication. (7.2) Furthermore, according to PCP and CHWs, having multiple concurrent prescribers, or changing prescribers, acted as a barrier to adherence, due to resultant disintegration of care. Uptake of counter-reference in practice and an integrated EHR system, was viewed as a key strategy to mitigate this problem. (7.3) Mental health problems were felt to be interfering with treatment of chronic physical disorders; anxiety and depression were both reported to limit self-management efforts and adherence to treatment, by reducing patient’s motivation and capability (e.g., obtaining medication and compliance with dietary recommendations). (7.4) Another PCP-related reason were difficulties with following instructions, according to HCP related to forgetfulness and understanding, as such increasing with low health literacy/illiteracy and polypharmacy. (7.5) Amongst reasons underlying discontinuation were also patients’ beliefs about ineffectiveness or negative consequences of medication use (particularly antidepressants) and (7.6) even religious beliefs (which reportedly play an important role in many PCP’ lives). (7.7) Interviewees mentioned various aspects of medication-related burden they experienced disrupting their daily lives (routine and lifestyle).

*(7*.*1)* ‘‘*We have many difficulties with the patients arriving here and are hypertensive*, *diabetic*. *The patient knows he/she needs medication because cannot do without it*. *Arrives here on the last day the medication is finishing and wants a prescription*. *We have a lot of difficulty with that*.*’’ (RN-M 6 –mixed FHS & BHU*, *female)*

*(7*.*2)* “*This business of changing doctors today*, *changing doctors tomorrow*. *In the end*, *you are in troubles with everyone*. *Isn’t that so*? *Because you are going today*, *then you express everything to a doctor*. *I even went there*, *talked to Dr*. *[name 1] and explained everything to him*, *okay*. *He gave me what [medication] was to be taken*, *everything*. *Then I went back there*, *it was no longer Dr*. *[name 1]*, *the other doctor was there already*. *Now is no longer that doctor*, *now is a she doctor*. *Are you understanding how it works*? *So you can never deal with anyone*, *because then it becomes that snowball*, *neither one nor the other*. *So look*, *I have seen so many doctors*, *that I don’t even know how to explain*. *It has been many doctors already*.*” (PCP 12 –FHS*, *female)*

*(7*.*3) ‘*‘*Sometimes you feel like*: *‘‘Oh my*, *why me*!? *With so many bad people [in the world]*, *right*?*” So*, *I had that worry*. *I said to myself*: *‘‘you are depressed”*. *My pressure doesn’t go down*, *oh*, *I can’t eat this*, *I can’t eat that*, *because my glucose would go up*. *There was a time when I was like that*, *you know*? *I used to say*: *‘‘Wow*, *why with me*?*” Damn*, *right*? *I wanted it so much*, *I don’t even know what*. *Then I relaxed*. *I said*: *‘‘no*! *If it has to be*, *it will be*. *Let’s take care and that’s it*. *But I think there are people who get even worse*, *right*? *And the person can sometimes no longer succeed*, *that is*, *to get rid of this depression in relation to glucose and*, *and hypertension*, *why*? *Yeah*, *they won’t be able to improve because they focus on that [feeling]*, *right*? *Seemingly it depends on this*, *to be able to live*. *You focus on that*, *you’re not going any further*, *right*?*” (PCP 17 –FHS*, *female)*

*‘*‘*I think the emotional illness problem makes therapy even more difficult because many times people associate their discouragement with the lack of effect*, *that they don’t get better*.*” (RN-M 3 –FHS*, *female)*

*(7*.*4)*‘‘*I take diabetes medicine*, *blood pressure*, *and there are these medicines here that I don’t know how to take*, *only for that I stopped*. *I can’t manage*, *right*. *I can’t manage**”*. *(PCP 5 –mixed FHS & BHU*, *female)*

“*I have another patient in my area*, *he can’t read*. *He can read only little and his wife can’t read at all and we have done several jobs with him*. *[*…*] He confuses things because he has hypertension*, *but [also] he has a heart problem*, *he has depression*, *and he receives treatment not only here at the [FHS] unit’‘ (CHW 11 –FHS*, *female)*

*(7*.*5)* “*When they told me it was fibromyalgia*, *that they couldn’t do anything there*, *that I was supposed to come back [follow-up]*, *continue with the post [attending the FHS unit]*, *I went*! *Then they passed Amytril [Amitriptilyne; antidepressant*, *anxiolytic*, *anti-pain drug] to take*, *right*? *[Amitriptyline]*, *and that’s it*. *Then I went to the appointments*, *I spoke*, *but (*..*) Nobody did anything*! *So*, *in terms of what I’m talking about*, *of doing nothing (*..*) I spoke about pains*, *everything*. *There have been people I had to listen to tellign me to learn to live with pain*, *right*?! *So I gave up for good*. *So I’m almost don’t*, *don’t chase after*. *I will not lie [to you]*, *I will tell the truth*! *(PCP 12—FHS*, *female)*

*(7*.*6)* “*That faith*. *I believe in God*. *God will heal me*. *I’m sure I’m going to get out of this here*. *I ask God*: *“Lord*, *help me*, *take this medicine [a lot of medicines] from me”*.*” (PCP 9 –FHS*, *female)*

*(7*.*7)* “*Yesterday I didn’t take [my psychotropic medication]*, *today I didn’t*, *because yesterday I drank [alcohol]*, *so I didn’t take it (*..*) It’s bad*, *right*?*” (PCP 19 –FHS with TSSP*, *female*)

“*The medications themselves*, *often it’s a matter of having to take them at the right time*, *sometimes they don’t [take them] because […] some habits will have to be changed*, *some [daily] routines will have to be changed*, *the fact of having to come to [see] the doctor more often*. *’’ (RN-M 3 –FHS*, *female)*

### 8) Most challenging health behavior change goals

#### 8.1. Lifestyle changes

When asked about what treatment goals seem most challenging, HCP univocally declared lifestyle changes; and this view echoed patients’ descriptions of the substantial burden of following their HCP advice on physical activity and special diets. (8.1.1) HCP felt that some patients have no intentions to change their behavior, even when faced with serious preventable consequences for health. Indeed, some PCP had exhibited an indifferent demeanour when prompted by an interviewer to talk about lifestyle changes that they were advised to make. (8.1.2) From HCP’ perspective, a lack of understanding of the effect of lifestyle change presents a barrier to conveying the importance of adopting healthier lifestyle changes. This echoes patients’ description of how successfull descriptions of their healthcare problem aided their decision to apply lifestyle changes. (8.1.3) Both HCP and PCP felt that patients face numerous challenges in their efforts to implement desired changes in lifestyle behavior. For example, the burden of long-term condition-induced need for lifestyle habit adjustments. (8.1.4) This problem being most apparent with sugar intake, which was viewed as deeply rooted in cultural norms, especially practices of excess sugar sweets consumption, and often discussed in relation to its consequent difficulties with breaking emotional ties to sugar. As such, it was reported by patients and recognised by HCP that changing dietary habits in social environments where those habits are common seems exceedingly difficult for patients. (8.1.5) Accordingly, some patients reported how having someone to accompany them in lifestyle changes acted as an enabler. (8.1.6) Lastly, HCP and PCP reported environmental context and resources, specifically lack of time and financial resources, adequate space, work schedule, often acting as practical barriers to lifestyle change.

*(8*.*1*.*1)* ‘‘*They [patients] prefer to take medicine rather than to remove salt [from their diet]*, *or to do physical activity*, *you know*? *And diabetes the same thing*. *What can we do*?*” (RN-M 14 –BHU*, *female)*

*(8*.*1*.*2)* ‘*‘I think they only know about pharmacological treatment*. *They are a lot like that*, *it’s medication*, *medication [*…*] When you talk about the importance of physical activity*, *many think it’s a lie*. *Many think that it’s enough to take the medication to be allowed to eat whatever they want*. *It takes a long time to wake up to the reality of having to go on a diet*, *it takes a long time*, *it is not an easy thing*.*” (RN-M 3 –FHS*, *female)*

*(8*.*1*.*3)* “*She [a doctor] wants me to walk*, *but I can’t walk*. *I have a walker and there are days when I have to walk inside the house with a walker*. *I can’t even walk alone*. *I’m afraid of falling because I’m fat*. *So I am very afraid of falling and breaking something*, *and then having to stay in bed*, *which is my dread*, *my daughters will not be able to take care of me*. *Only I know how difficult it is*.*" (PCP 1 –FHS*, *female)*

*(8*.*1*.*4)* ’’*I think that many people get really depressed*, *many get [depressed] and I think the fact that they can’t follow a diet*, *they can’t*, *is because it’s like you restrict the best thing these people have*, *which is to eat*, *you ever thought about it*? *’’ (RN-M 14 –BHU*, *female)*

*‘‘There is an association between food and love, it exists in our culture, in our way. I have, for example, patients who say: "the only way she gives us love is that cake, that food she makes". There is a cultural association that food is love. And sugar is also associated with that.” (manager of mental health service (MMHS) 3—female)*

*(8*.*1*.*5)* “*Even during the weekend*, *I go down to [a name of a place] to go hiking*, *but it’s not the same*. *Is very bad*. *Now*, *with the group*, *it is totally different*. *[*..*] getting out of that little world I was in and doing the group walk is how my depression improved*.*” (PCP 8 –mixed FHS & BHU*, *female)*

*(8*.*1*.*6)* “*The poorest population does not have access to a diverse diet so I can’t*, *I can’t even try to demand a diet from that [kind of] patients*, *that is*, *fruits and vegetables*, *since he/she doesn’t have the money to buy it*. *He/she has money for the basics*. *[I tell the patient*:*] “Oh*, *I wish you were exercising”*, *but he/she works all day*. *So sometimes he doesn’t have time to exercise more*, *so many [patients] come and say*: *“oh*, *but I already work all day*, *I want to go home*, *I’m tired”*.*” (PHY 9 –BHU*, *female)*

#### 8.2. Depression and anxiety drugs abuse and misuse

Most HCP reported the alarming problem of wide-spread incorrect use of antidepressants and anxiolytics by PCP. (8.2.1) PHC and mental health staff felt that patients often ‘intentionally’ take prescribed antidepressants incorrectly, namely skipping, or missing doses, or taking their medication at irregular intervals. Detailing provided by some PCP confirm skipping and missing doses, but the provided motives included fear of dependency and interfering co-existing healthcare problems. (8.2.2) HCP expressed their concerns over common dependency on antidepressants and anxiolytics, which often originate from abuse and overuse. Accordingly, some descriptions provided by PCP indicate psychotropic drug misuse and subequent dependency, of which they seemed fully cognizant at that point. (8.2.3) HCP were particularly alarmed with benzodiazepines being often used to manage sleep problems, which from their experience was a form of prescription drug abuse. There is evidence for that in interviews with PCP, for all of whom this was a reason for concern. (8.2.4) When prompted to speculate about the origins of this problem, several physicians attributed it to the negative social influences, such behavioural modeling, or even encouragement and facilitation by family and neighbours. However, while some PCP indeed mentioned their decisions to seek care for mental health problems being influenced by people in their social network, once they experienced negative effects of psychotropic mediction use/misuse, they all seemed aware of or even felt negative about psychotropic medication use. Therefore, the narrative emerging from PCP’ stories is that of misuse related to incomplete understanding of psychotropic medication effects, beliefs about negative consequences of their use, personal struggle with overcoming co-dependancy and possibly suboptimal supervision by prescribers.

*(8*.*2*.*1)* ‘‘*A patient also*, *sort of*, *doesn’t understand the treatment [for depression]*. *There are patients who think that the medication for depression*, *for psychiatry is ‘just taking medication’*, *and also taking it when you are not feeling well*. *[*…*] Then*: *“Have you taken your medication*?*”*, *“Ah*, *the day I see that I’m not well*, *I take it”*. *I mean*, *there is no point in taking fluoxetine one day*.*” (PHY 8 –FHS*, *male)*

*(8*.*2*.*2)* “*No*, *this [an antidepressant] not*, *I just take one diazepam from there [a MHCC]*, *right*? *From there [I take] only diazepam*. *Only*, *now the stronger ones do not*, *diazepam helps to sleep*, *but it’s not helping me*, *I am still not able to sleep*. *I didn’t sleep this night*. *[…] There are people there who take those strong medicines that turn them into a parasite*, *it is like with (*..*)*, *I don’t know*, *like they have medicine there that makes you crazy*, *you are not crazy*, *but that leaves people walking around*. *No*, *I already took these*. *I already did*. *But I didn’t take it afterwards*, *I got bad*. *I told you I was unable to walk at all*, *I had to go to the emergency department (*..*) in an ambulance*.*” (PCP 19—FHS with TSSP*, *female)*

*(8*.*2*.*3)* “*I couldn’t sleep without the medicine [benzodiazepines]*, *do you understand*? *Wow*, *I didn’t sleep all night*, *so I’m getting addicted on it*, *so I think it’s good to see if I can get it off*.*” (PCP 15 –BHU*, *female)*

*(8*.*2*.*4*.*)* “*Out of the blue I felt my heart racing*, *so I saw that it was indeed anxiety*, *and so from then on I started to take a tranquilizer*, *I needed to take it […] And there was a time when I got really doped*. *The psychologist yes*, *I miss having one*. *But*, *not (*..*) not a psychiatrist*, *no*. *Because thanks God I stopped taking that medicine*. *I managed to do it*. *God took me out of it*. *I stopped little by little*.*” (PCP 12 –FHS*, *female)*

’’*For depression*, *a patient wants to know about [specific psychotropic] medication*, *because it’s like this*: *he took someone’s medication*, *he/she had that thing where can’t sleep and there is always someone who gave a medication at home*, *who gave it to that person*. *"Ah*, *that medication was very good for me*, *doctor*!*"*, *You know*? *(PHY 8 –FHS*, *male)*

#### 8.3. Insulin injections

HCP extensively discussed their experiences with managing the burden of insulin therapy. (8.3.1) In their experiences, patients often resist the use of insulin injections. They speculated that this resistance is mostly related to patients’ beliefs about the implications of insulin injections for one’s sense of self, that it represents ‘being very sick‘ (even dying), where by refusing injections they were resisting illness identity (i.e., the degree to which a condition is integrated into someone’s identity). Accordingly, some of the interviewed patients expressed their fears about the implications of insulin therapy and described how unpleasant it initially was. (8.3.2) FHS team members often reside to mitigating this problem by involving several team members (e.g., nurses and pharmacists) in the process of educating, monitoring and making insulin injections. (8.3.3) Interviewed BHU physicians, on the other hand, stated that they were often unable to initiate and monitor insulin therapy and were forced to refer a patient to an endocrinologist.

*(8*.*3*.*1)*"*When a diabetic gets to the point of having to take insulin*, *holy mother*, *they are very difficult*, *very resistant*, *a very resistant person*.*" (CHW 7 –FHS*, *female)*

*(8*.*3*.*2)*“*Some cases*, *more complicated ones*, *that we are unable to monitor*, *when a patient uses insulin*, *for example*, *at home and nobody wants to apply it and he/she [the patient] does not know how to apply it alone*, *they [PHC staff] give the medication at the clinic [the mixed FHS-BHU unit]*. *Sometimes*, *there have been some patients that I asked to take medication at the clinic*. *They go there in the morning and at night*. *It is not ideal*, *you know*, *the right thing is to try to make them take it at their house*, *but when there is no way*, *it is complicating his illness and he is not taking the medication properly*, *we do it that way*. *Then the nursing staff do their medication*.*” (PHY 6 –mixed FHS & BHU*, *male)*

*(8*.*3*.*3) ’*’*The same thing*, *diabetes*, *many times we try to keep them at the unit as much as possible progressing with the oral medication*, *then the insulins come in*, *and then we have to refer to an endocrinologist*.*’‘ (PHY 9 –BHU*, *female)*

### 9) Main motives for initiation or maintenance of treatment

(9.1) PCP explained how they perfomed their behavior if they had at least one sustained initation/maintenance motive, that is they enjoyed engaging in the behavior, they were satisfied with outcomes or the behavior was congruent with their social role/identity and beliefs about consequences. (9.2) PCP also described being able to successfully maintain desired behavior if they monitored and regulated the newly adopted actions and have effective strategies to overcome barriers to their performance.

*(9*.*1*.*)* ‘‘*The doctor told me*, *my doctor Dr [name] said to me*: *“People who do constant walking don’t even have to take medicine*, *right*? *[*…*] I said to myself*: *“I’m going to take an hour and go for a walk*, *even if it’s only in my neighourhood*, *right*? *It was good*, *I felt really good*. *I felt really good*, *so I carry on with it*.*” (PCP 16 –FHS*, *female)*

*(9*.*2)* ‘‘*Today I take Gliclazide [medication for diabetes] in the morning*, *one and a half pills*. *I take*, *Glifage [metformin; medication for diabetes] and the SSRI [selective serotonin reuptake inhibitor; antidepressant]*, *it is two after lunch*, *two after dinner*. *I policed myself*, *regarding food*. *[*…*] But it is so difficult today*, *right*? *With so much good stuff that exists today*, *but you* *have to* *police yourself*. *And I ‘closed my mouth’ [resisted]*, *as they say*, *I learned new habits*, *I managed to lose twelve kilos*, *right*?*” (PCP 17 –FHS*, *female)*

### 10) Methods deployed to improve treatment adherence

HCP described deploying a range of methods to improve treatment, especially medicine adherence. (10.1) Enhancing involvement of patients’ social network was widely regarded by HCP an important method, particularly for illiterate and elderly patients. Indeed, PCP widely stressed and detailed the pivotal involvement of support from social networks—including family members, peers and neighbours—for treatment adherence. They found that members of their social networks were very helpful by: actively encouraging them to manage their health problems; actively assisting with correct use of medication (e.g., helping with medication and taking the capillary blood glucose measurement) and even providing practical support in the form of mitigating financial barriers (e.g., money for medication or food). (10.2) FHS teams exclusively reported illiterate and elderly patients lacking social support being offered extra attention from healthcare providers, in the form of more frequent contact, intense guidance and collaborative care. Accordingly, to improve treatment adherence, patients stressed a need for more frequent contacts with their physicians (through shorter-interval and longer-duration follow-up appointments), quicker and easier access to HCP’s advice on medication (via text messaging or phone) and enhanced continuity of care (having a physician of reference and a trusted ongoing relationship with him/her). Some PCP also reported finding useful a direct feedback or criticism from physicians on their treatment compliance. (10.3) CHWs described their assistance with organising patients’ medication using creative ideas and solutions (e.g., drawings, colours, counting out loud, leaving pill boxes for members of the same family in distinct locations in a house). (10.4) HCP in both BHU and FHS units, also discussed direct strategies of available mitigating problems with access to prescription medication, by scheduling prescription renewal and using only drugs available through pharmaceutical assistance programs, which resonate with PCP’ descriptions. FHS team members described additionally arranging prescriptions with any available prescriber or as a last resort enabling social service assistance.

*(10*.*1)* “*Then*, *I went after my niece because my children don’t visit me*, *they work*, *and they don’t allow me to work*. *Then she [the niece] said*: *"No*, *aunt*, *you can let me come and give the medication to Ma’am the right way"*. *(PCP 9 –FHS*, *female)*

*’*’*There are patients whose daughter sometimes comes*, *a father comes*, *if there is someone who cares but doesn’t separate [medication]*, *then the nurse separates*. *[But] usually we look for someone in the family who can do this for the person*, *a father*, *a brother*, *a neighbour*, *a son*.*’’ (CHW 5 –FHS*, *female)*

*(10*.*2)* “*I think they [a BHU team] should be more on top of it*, *right*, *because every six months*, *what if something suddenly happens*, *right*? *You should give more space [for check-ups]*, *right*? *Imagine you take insulin for six months and insulin is not working*, *and you need to you wait to see a doctor to tell her*, *right*? *Yeah*, *the time needs to be shorter than that*, *right*? *(PCP 15—BHU*, *female)*

*’’It depends on the patient, same thing if the patient is bedridden, domiciled and such, can’t move, and we can’t get anyone to do that for him, like a son, a neighbour and such, then we do and if the patient adheres to the treatment, the nurse is available to separate the medications.” (CHW 5 –FHS, female)*

*(10*.*3)* “*So [I will know*, *if he is taking the medication]*, *because we also do and organize it*. *There are patients who use a lot of medicines*, *so we organise them by bottle*. *Then we put a glove*, *sun*, *soup plate*, *to indicate*, *right*. *The hours and we paint the caps*. *Let’s suppose*, *the little red is a pressure medicine; the blue whiting is diuretic or for the heart*, *you know*. *We separate by colour and time*, *like that*, *with the drawings*.*” (CHW 8 –FHS with TSSP*, *male)*

*(10*.*4)* “*Convincing him/her [a patient] to use the medication*, *he/she then go on an uses the medication*, *but then we have the limitation of the medication available on the [healthcare] network*. *A patient here has a [certain] socioeconomic level*, *so we are unable to use the medications that are*, *let’s say*, *the most recommended for that disease or that are (*..*) that have the best result*, *that studies show that had the best results*, *that patient does not need taking as many times*, *the patient can control something [disorder] so that it gets closer to the normal*. *There’s no way*, *we work with medication that is given on the [healthcare] network*. *If he/she [patient] often doesn’t find it*, *he doesn’t use it so it’s useless*, *I have to know what I’m going to prescribe*.*” (PHY 9 –BHU*, *female)*

“*It depends*, *sometimes we reach out to social services*, *because we have had cases like this [without access to medication] most of the times when they run out of the medication they is unavailable through the [health system] network*, *they buy it themselves*, *it’s like two hundred*, *three hundred reais per medication*, *it’s a lot*.*” (CHW 5 –FHS*, *female)*

## Discussion

Our study comprehensively demonstrated biopsychosocial complexities inherent to management of mental-physical MM in Brazilian PHC, which induce treatment burden experienced by both patients and their caregivers. This presents a considerable challenge for treatment adherence. This is the first such research in the context of physical-mental MM, in a LMIC with a fully decentralized public healthcare system.

### Comparison with existing literature

Many of the reported challenges in MM care, were previously reported by HCP [57] and PCP [32], predominantly in high income countries. Including, insufficient consultation time [58, 59]; issues with depersonalised care and form or level of information provided by HCP [59]; poorly coordinated or fragmented care [58]; and limited information exchange between HCP [58, 59]. Issues with deployment of integrated EHR–a key component to improved care coordination and information exchange–has been reported worldwide [60, 61]. Our findings support the recognised importance of medication burden, specifically dose frequency and regimen complexity for treatment adherence [62] and efforts to minimize that burden by reducing dosage frequency [63]. Burdens of understanding the condition, juggling, monitoring and adjusting treatments, efforts to engage with others for support as well as financial and time burden, have been previously highlighted [5]. The critical role of CHW in treatment monitoring, reiterates previously described importance of these HCP in LMICs for continuity, completeness of care [64] and even decreased rates of physical [65, 66] and mental health disorders in PHC populations in Brazil [67] and India [68]. Related to that—the role of FHS model—was previously found to be associated with greater patient satisfaction and improved outcomes in people with MM [38, 39]; our work reiterates that and also shows that a close trusted relationship fostered through this PHC model results in a good understanding of barriers PCP face. The findings on interference of mental disorders with treatment adherence for other chronic conditions also exemplifies previous descriptions [69, 70]. The importance of illness acceptance and an individualised approach to patients in communication and care planning, echoes previous postulates for exploration of personal meaning of health [33, 71]; adoption of a broader care strategy inclusive of pluralistic and personalised capabilities [72–74]; and treatment initiation through elicitation of personal motives for change through self-reflection and provision of individualized information [9, 16, 33–35, 75].

### Implications for research and practice

The results of this research highlight the value of adopting in Brazil the biopsychosocial [76–78] and person-centred approaches [79] in MM care. A shared critical component of both is a “whole-person perspective” approach (integrating bio, psycho and social dimensions) [80], in practice involving exploring patients’ ideas and feelings; and elicitation of defining personal objectives and strategies to achieve them [81]. It is still unknown how to operationalize this in practice in Brazil, but there are several candidate models and strategies that are compatible, which have proved effective in other countries. Starting with a broader model of care, such as the Chronic Care Model (CCM) [82], which incorporates practical suggestions for the development of its six healthcare areas (i.e., self-management support, delivery system design, decision support, clinical information systems, organization of health care, and community), that can be tailored to any country context. Training in Patient Centred Medicine (CM)—compatible with CCM [83]–can help HCP to systematically deliver four care tasks (i.e., exploring health, disease and the ilness experience; understanding the whole person; finding common ground; and enhancing the patient-clinician relationship) [84]. Care-Plus (CP) [85] and Ariadne Principles (AP) [86], offer specific strategies, studied in other countries, to effect better bonds and communication between professionals and patients with MM. Motivational Interviewing, a strategy with proven effectiveness in the Brazilian PHC [87], can help to train HCP to co-create care plans with PCP to meet their biopsychosocial needs [88]. Motivational interviewing can also be integrated with other psychological interventions useful in PHC, like Acceptance and Commitment Therapy [89], Behavioral Activation [90] and Problem-Solving Therapy [91].

Evidence from developed countries suggests that interventions to improve patient behavior related to adherence outcomes are suboptimal. A systematic review showed that interventions may only slightly improve medication adherence (low certainty evidence) and probably slightly improves patient‐related health behaviors, such as adherence to diet and exercise (moderate certainty evidence) [17]. Organization-level interventions, such as integrated care approaches, show the most promising potential in addressing the challenge of adherence management in mental disorders in LMICs, where in two studies adherence management were one of the active ingredients of the integrated care model [92], but in general a comprehensive model that better accounts for the complexities (e.g., complications, frailty or vulnerability) related to MM is needed [93–95].

As for the complexities in Brazil, we showed that in our study population and context, complexities of mental-physical MM care in PHC routinely go beyond the combination of different conditions and treatments interacting in intricate ways, into effects of previous social and health experiences (e.g., treatment burden, quality of PCP-HCP relationships), demographics (e.g., a lack of resources, low health literacy), and social capital (e.g., support from family and neighbours). This was described previously as ‘health complexity’ [23]. Sturmberg et al. [18] recently argued for a person-focused care based in complexity science as a transformational lense to MM care. The authors proposed a coherent approach to understanding and managing MM, by integrating biological, biographical, and contextual factors, which may be well suited to guide development of culturally appropriate integration of care in our study population.

While different culturally appropriate interventions may be needed for different MM groups [96], our study suggests that future efforts to improve care effectiveness, accessibility and equitability in the studied and alike populations will face the challenge of optimizing care planning strategies, in a way that prioritizes motivation-matched person-centred treatment goals using individualized communication, which might in turn improve patient acceptance and motivation and increase adherence to treatment, with the ultimate aim of potentially improving personal outcomes. While regrettably participant involvement in the research cycle is still not a standard practice in Brazil, we suggest working collaboratively with patients and frontline staff when developing those future interventions, with plenty of excellent frameworks available to support it [97]. Our study also illustrates the importance of working towards overcoming practical barriers, such as technological challenges to more effective health information systems (HISs) from which data and information can be derived to improve service outcomes [98, 99] and communication (e.g., telemedicine) [100].

### Study strengths and limitations

The present study involved a large and heterogenous sample of HCP and PCP using rigorous methods to ensure comprehensiveness. To maximize its relevance and impact, the study was HCP-led. To ensure trustworthiness and validity of findings we applied data source and analytical triangulation, reflexivity, clear exposition of methods of data collection and analysis, attention to negative cases and incorporation of different perspectives [101]. Mindful that it was typical for the studied population have limited education, we piloted and adapted the topic guide to overcome communication challenges, inarticulateness, frame of reference and the concept of time [51]; and we used in-depth interviews and carefully matched interviewers with interviewees to facilitate the voice and agency [44, 102].

An implication of focusing on PHC for transferability and relevance, was that the results mostly concern adherence to medication and lifestyle changes, the first line treatment offered by PHC teams for mental-physical MM. To ensure relevance to the study setting, we intentionally selected for eligibility screening disease or conditions most common in Brazil and mainly managed in PHC. In practice, interviewees frequently chose to use examples from diabetes and hypertension care. As such, transferability of findings on condition-specific treatment may be limited (e.g., emotional ties with sugar or insulin injections), as oppose to generic aspects of quality care or care pathways discussed by the interviewees. Given the size of Brazil, its demographic and economic heterogeneity and fully decentralized character of its healthcare system, the perspectives presented here may vary from other Brazil regions.

The interviewed PCP represent one of the most vulnerable groups of PHC patients—elderly women, urban dwellers, public healthcare attendees, without any or limited education (some even illiterate), with numerous debilitating conditions. Those features together with living in a cultural context where a doctor holds an expert power, and the greater volume of HCP data, compel us to continously resist the urge to put at forefront the more articulate voice of HCP. To reduce the risk of misinterpretations and some blanks being left to be filled in future qualitative research on this population, we suggest methodological triangulation [44] (e.g., interviews with observations or an ethnographic approach). Translation and cultural adaptation of validated burden questionnaires specific to MM could offer new opportunities to quantify at scale most burdensome aspects of treatment or even aid care monitoring [103].

## Conclusions

People with mental-physical MM in Brazil are among the most medically and socially vulnerable people in the world, but the optimal solution to preserve their ongoing care is very complex. This study fits in the existing literature, showing patient and caregiver treatment burden associated with MM care, and importance of person-centred and biopsychosocial approaches to effect treatment adherence, most apparent in patients with low health literacy and no social support. The complex interplay of health and socioeconomic factors in Brazilian PHC, supports approaching the understanding and management of MM through a ‘complexity lens’. Daily efforts of PHC teams to effect changes in treatment adherence reveal possible short and mid-term solutions, such as reduced dosage frequency, robust care planning and integrated HIS and telemedicine.

## Supporting information

S1 Appendix Exemplary interview topic guides (English & Portuguese). (PDF)

S2 Appendix The thematic tree & exemplary quotes. (PDF)
