# Supplementary material for: Mental-physical multimorbidity treatment adherence challenges in Brazilian primary care: A qualitative study with patients and their healthcare providers
Source: PLoS One. 2021 May 13;16(5):e0251320. doi: 10.1371/journal.pone.0251320 (PMC8118469; doi:10.1371/journal.pone.0251320)
Supplement: S1 Appendix — (PDF) [file pone.0251320.s001.pdf]

S1 Appendix

Exemplary Interview Topic Guides (English & Portuguese)

A conversational style of interviewing was adopted, to encourage comfortable and fluent dialogue rich in detail, while using a semi-structured interview topic guide as a reference to ensure that all key topics were covered. We have attempted to cover each key topic with each interviewee. Consistent with good qualitative research practice, main questions and prompts [text in square brackets in grey font] varied in each interview, the former dependent on whether the participant already spontaneously covered that topic or not and the latter dependant on the participant's experience and their opening narrative.

English versions

Interviews with Physicians

[Topic guide questions were tailored to specific healthcare professional group, according to relevance]

- Opening question: Could you describe your role/ involvement in care for people with diabetes, hypertension, heart disease or arthritis/arthrosis in the primary care unit where you work?
- There are people who have two or more of these chronic illnesses at the same time (for example, diabetes, hypertension, heart disease or arthritis). Is your involvement in care for these people any different compared to your involvement in caring for people who have only one of these chronic diseases?
- Some health professionals say that they have observed that some patients do not feel comfortable when they tell them their problems and difficulties. Has this situation happened to you? [In your experience, what type of patient is most comfortable telling the problems and difficulties and which one is least comfortable?]
- Some health professionals say that they have often had difficulty understanding what patients wanted to ask or their explanations of what was happening to them. Has this situation happened to you? [If yes, is it common? For example, some people think that elderly people / people with little education may have difficulties in communication, and others do not. What are your experiences on this?]
- How do you get to know the current life situation of each of your patients, that is, for example, where he/she lives, what is his family like, if he works or not, what is his financial

situation, if he is religious? [If so, how do you know this information? If not, why are you unable to know this information?]

- How do you get to know details of the life history of each patient you follow, that is, for example, where he was born as he was to the family he grew up in the places where he lived, what he studied? [If so, how do you know this information? If not, why are you unable to know this information?]
- How do you get to know the complete health history of each patient you care for, that is, what happened in the appointments he has already done in different health services and with different professionals? [If so, how do you get to know this information? If not, why can't you?]
- How do you know if every patient you are seeing who has diabetes, hypertension, heart disease or arthritis is getting help for any of these chronic illnesses at other health care services other than the one where you work? [If so, can you give me more details on how you got to know? If not, can you give me more details on why you can't find out?]
- How do you know which medications have been prescribed for each of your patient who has a chronic illness, including those medications that have been prescribed in health services other than the health service here, where you work? [If so, can you give me more details on how you got to know? If not, can you give more details on why you can't find out?]
- How do you know if the patients you care for are taking the medications they were prescribed correctly?
- How do you find out which of the patients you care for have diabetes, hypertension, heart disease or arthritis? [In your experience, what are the best diagnostic methods for hypertension, diabetes, heart disease, arthritis to use in Primary Care?]
- When you have to decide how you decide which treatment is best for a patient with one or more of these chronic diseases? [For example, how do you decide to advise a patient to take specific medications, or to do some kind of physical activity, or to eat certain types of foods? How is your use of the “Primary Care Notebooks”, from the Ministry of Health, to help you plan and decide what is the best treatment for your patients?]
- What treatments or ways to help treat are offered for hypertension, diabetes, heart disease, arthritis / arthrosis in your care unit?
- Of the treatments that are offered for these chronic illnesses in this health service, which ones do you think help the patients a lot and which ones do you think do not help that

much? [Why do you think that? Do you think there is a combination of treatments that is especially good for helping patients with these chronic illnesses?]

- If you had a choice, what treatment - or treatments - would you like to offer to patients with diabetes, hypertension, heart disease or arthritis / arthrosis?
- In you experience, people with hypertension, diabetes, heart disease or arthritis, also have co-exiting, emotional problems such as depression or anxiety? [It is common?]
- Do you think that hypertension, diabetes, heart disease or arthritis can cause depression and anxiety? [If so, give more details on how you think chronic illnesses can trigger emotional problems.]
- Do you think that depression and anxiety can help cause hypertension, diabetes, heart disease, arthritis or osteoarthritis? [If so, give more details on how you think these emotional problems can help to cause chronic illness.]
- What else do you think can help cause depression and anxiety?
- What is your involvement in the care of people who have hypertension / diabetes / heart disease / arthritis or osteoarthritis and also have depression and / or anxiety at the same time? [Can you give more details on this?]
- Do you think that the way each patient deals emotionally with these chronic illnesses - for example, if hypertension or diabetes makes the patient more sad, nervous or, on the contrary, he can keep himself happy and calm - can make a difference, for the treatment of these chronic diseases to work more or less well? [Tell me more, give more details, what do you think about it]
- Can you ask patients with these chronic illnesses how they feel emotionally? For example, can you ask if they are sad, discouraged, down, or on the contrary if they are excited, in a good mood, happy? [If yes, give more details on how you ask about it. How do patients respond to being asked that way about their emotional state? How do you feel asking about emotional problems? If not, give more details as to why you can't ask about it]
- How do you decide when to ask a patient questions about his or her emotional state - that is, for example, ask if he is feeling sadder, nervous, in a low mood or, on the contrary, if he is happier, in a good mood ?
- Some health professionals say that, in their experience, older people or men have more difficulty talking about emotional problems. In your experience as a professional in this primary care service, does this happen? [If yes, give more details about your experience]

- How did you deal with these difficulties with talking about emotional problems? [How do you think it would be a good way to deal with these difficulties in talking about emotional problems with the elderly and men?]
- Do you use any specific method or strategy to assess the severity of symptoms of depression and anxiety, in people you attend in this health service who have, at the same time, hypertension, diabetes, heart disease, arthritis / arthrosis? [If so, which one? If not, why? If not, how do you find out which patients you see who have depression and anxiety?]
- Some health professionals report difficulties in assessing symptoms of depression and / or anxiety in people with hypertension, diabetes, heart disease, arthritis / arthrosis. How's that for you? Have you ever encountered this kind of difficulty in your work? [If so, what were the difficulties?]
- How did you deal with these difficulties? [How do you think it would be a good way to deal with these difficulties to assess symptoms of depression and / or anxiety in people with chronic illnesses?]
- How do you know, for each patient you attend, if medications were received who you prescribed to treat depression and / or anxiety? [Can you give me more details about how you get to know (or why you don't get to know)? If not, what do you think about it?]
- How do you know, for each patient you see, if any other health service has been prescribed medication to treat depression and / or anxiety? [Can you give me more details about how you know (or why you can't know)? If not, what do you think about it?]
- How do you know if each patient is taking the medication prescribed to treat anxiety and / or depression correctly?
- How do you know, for each patient you attend, if he received any help, other than medication, to treat emotional problems such as depression and / or anxiety, right here in this health service? [Can you give me more details about how you get to know (or why you don't get to know)?]
- How do you know, for each patient you see, if he/she received any help, other than medication, to treat depression and/or anxiety, in another health service? [Can you give me more details about how you get to know (or why you don't get to know)?]
- What ways of helping people with depression and/or anxiety are offered at the health service where you work?
- In your experience, which forms of treatment work and which forms of treatment do not work well, to help people improve from depression and/or anxiety?

- If you had a choice, what kind of help would you like to offer to your patients to treat depression and anxiety that you would? [Why, this one?]
- In your experience, what ways do your patients prefer to treat depression and/or anxiety? [For example, do some patients prefer to take medication rather than receive some other type of help to treat depression and/or anxiety? Do some patients prefer to receive help of any kind that does not include taking medication to treat depression and /or anxiety?]
- When a patient of yours, needs a referral for treatment of chronic illness, can you or someone in your primary care team talk to them about the specialised services in which they could be seen? [If so, how do you or someone on your team manage to make this conversation? If not, why can't you or someone on your team make this conversation?]
- When your patients need referrals to treat emotional problems, can you or someone on your primary care team talk to them about specialised mental health services that they could be seen to? [If so, how do you or someone on your team manage to make this conversation? If not, why can't you or someone on your team make this conversation?]
- Can you or another professional in the primary care service where you work help the patient to make an appointment or be admitted to other health services, if necessary? [If so, how can you or someone on your team help the patient with this? If not, why can't you or someone on your team help the patient with this?]
- Have you ever noticed that any of your patients experienced difficulties with getting care in other health services to treat hypertension, diabetes, heart disease, arthritis / arthrosis, even after you or another professional in this health service made a referral to these other services? [If so, what difficulties have you noticed? How did you deal with these difficulties?]
- Have you ever noticed that any of your patients experienced difficulties with getting care in a mental health service, even after you or another professional at this health service made a referral to these other services? [If so, what difficulties have you noticed? How did you deal with these difficulties?]
- When your patients are referred, can you or someone from this primary care service provide them with written information (a report, a reference form) to take to the specialist or specialised service? [If so, how do you or someone on your team do this? If not, why can't you or someone on your team do this?]
- After your patient has been consulted or admitted to a specialised service, do you or anyone in this primary care service receive a report, a counter-reference form, from that

specialised service? [If so, what is the quality of this counter-reference form? If not, why do you think they are unable to provide the counter-reference form?]

- Do you or someone in this primary care service talk to your patient about the results of this consultation or admission to the specialised service after it occurred? [If yes, talk more, give details, about these conversations? If not, why doesn't this conversation with the patient happen?]
- Thinking about the aspects of care we discussed, how would you describe your satisfaction with communication with other health professionals, including colleagues from this healthcare service and other health facilities on the treatment of their patients? [What works? What aspects need improvement?]
- Do you have any suggestion (s) about how communication with other health professionals who look after your patients could be improved?
- Can you recommend some strategies or ways to integrate treatments for chronic illnesses with treatments for depression and / or anxiety, which work here in your health service?
- Do you have any suggestion (s) on how the integration of these treatments for these two types of problems could be improved here in your health service? [For example, ideas about training health professionals or ideas about how to operate a system or equipment that facilitates communication within your health service and/or with other health services?]
- <u>Closing question:</u> In the context of our conversation, is there a topic that is important to you, but you haven't had a chance to speak?

Interviews with Patients

[Diabetes and depression are used for illustration purposes, but specific condition would vary from a patient to another]

- <u>Opening question:</u> When for the first time a doctor or other health professional told you that you have diabetes [Note: **OR OTHER CHRONIC DISEASE**, allow the patient to choose one/more, and amend the following questions accordingly]? [Who said that and in which health service?]
- In what health services you received care for diabetes? [Note: In the case of the person having passed by many services or to remember better, you can suggest trying to think about the last year]:

a. In which healthcare services?

- ☐ Primary care unit (BHU or FHS)
- ☐ Centre of Specialties, Public Polyclinics or PAM - Medical Assistance Post

- ☐ Emergency Care Unit
- ☐ Other type of Public Emergency Service (24 hours)
- ☐ Public hospital emergency room or emergency
- ☐ Public hospital / ambulatory
- ☐ Private practice or private clinic
- ☐ Ambulatory or office of a company or union
- ☐ Private hospital emergency room or emergency
- ☐ At home, with a doctor from the family health team
- ☐ At home, with a private doctor

b. **By which health professionals** were you treated for **diabetes?**

- ☐ doctor
- ☐ nursing staff
- ☐ community health worker
- ☐ physical therapist / occupational therapist
- ☐ physical educator
- ☐ nutritionist
- ☐ pharmacy
- ☐ social worker

c. **What care** or ways to help treat you received for **diabetes**:

- ☐ Medication
- ☐ Laboratory or imaging tests or complementary diagnostic tests (blood, urine, X-rays, etc.)
- ☐ Guidance on life habits
- ☐ Other [Which one?]

- Have you ever found it difficult to make an appointment or get treatment for diabetes? [What difficulties did you have? E.g: cost, distance to a service unit, opening hours, waiting time for a consultation at a specialized clinic or others]
- How did you managed to resolve those difficulties?
- Some people think that people with darker skin colour or a complicated financial situation may find it more difficult to access health services for diabetes, and others do not think that. What are you experiences with it?
- Among these service locations in which you were attended for diabetes, to which one you come back more often?
- When you come back in this service is it always the same doctor or nurse who takes care of your diabetes?

- In your opinion, is there a health professional or health service that knows you better as a person [that is, knows at least a little about the history of your entire life, about whom you live with, about your professional situation] and knows better history of your diabetes?
- There is a health care or health service that you consider to be responsible for your diabetes care?
- Have you ever had a hard time understanding what some health professional said about diabetes? [If so, what happened?]
- Do the health professionals who care for your diabetes know about you as a person, in addition to diabetes itself? For example, who do you live with, where do you work, if you have problems getting or paying for medicines to treat diabetes?
- During consultations with healthcare professionals related to your diabetes, do they give you enough time to talk about their concerns and their views on their problems with diabetes?
- Do healthcare professionals who care for your diabetes ask about your feelings? For example, if you are feeling sad, or nervous, or worried, or angry or with some other emotion? [Would you like to talk about the feelings, about your emotions, with the health professionals who care of your diabetes?]
- Have health professionals who care for your diabetes ever asked you what you would like to do to get better? [If yes, give more details on how this was done. If not, would you like to be more involved in making decisions about how to care for your diabetes? For example, I would like a doctor to discuss with you which way of helping to treat your diabetes (with medication/without medication) would be better for you, what aspects of your health would you like to improve (lessen pain/be more able to do the things in the house). If you say, "I just want to get better", ask what it means to him/her?]
- What do you think about ways to treat/manage diabetes (treatment, examination and advice) you receive?
- What helped and what did not help much to improve diabetes of things you receive or received? [Why?]
- If you could choose, what would be the ways to help treat the diabetes of your own?
- If you could choose, in terms of your diabetes, by whom you would like to be attended, in which health service of health and at which times? [Explore the person's attitude toward participation of different professionals in this process. For example, if the person says

would like to be seen by Doctor X, ask why? What do you think about the idea of nursing staff to treat your diabetes?]

- When was the first time that a doctor or other health professional told you that you had depression [Note: **OR ANXIETY,** check with the patient and allow to choose]? [Who said that?]

- In which health services did you receive care for depression? [Note: If the person has attended many services or can't remember well, you can suggest trying to remember only about the last year]:

d. **Which** healthcare service?

☐ Primary care unit (BHU/ESF)
☐ Center of Specialties, Public Polyclinics or PAM - Medical Assistance Post
☐ Emergency Care Unit
☐ Mental Health Community Centre (MHCC)
☐ Mental Health Outpatient Service
☐ Other type of Public Emergency Service (24 hours)
☐ Public hospital emergency room or emergency
☐ Psychiatric hospital
☐ Other public hospital / ambulatory
☐ Private practice or private clinic
☐ Ambulatory or office of a company or union
☐ Private hospital emergency room or emergency
☐ At home, with a doctor from the family health team
☐ At home, with a private doctor

e. **Which health care professionals have** you been treated for **depression**?

☐ doctor
☐ nursing
☐ agent community health
☐ psychologist
☐ psychiatrist
☐ occupational therapist
☐ social worker
☐ Others [Which one?]

f. **What care** / treatment did you receive for **depression**:

☐ Medication
☐ Psychotherapy [for example, talking about the problem with a psychologist]
☐ Guidance on life habits
☐ Others [Which one?]

- Do you feel comfortable talking about emotional problems with others?

- Whom do you usually talk to about your emotional problems? [Examples: family / friends / family doctor / psychiatrist / other health professionals/others?]

- Some people think that the diabetes [other] can cause depression or has an impact on depression. What do you think?
- In contrast, some people think that depression may help cause diabetes or have an impact on depression. What do you think?
- What else do you think can help trigger/aggravate depression?
- Since you found out you have diabetes, have you found it difficult to make an appointment or get treatment for depression? [If so, what difficulties did you have: cost, distance to a service unit, opening hours, waiting time for an appointment with a specialized service or others]
- How have you dealt with these difficulties?
- Some people think that people with darker skin colour or a complicated financial situation may find it more difficult to get help from health services to treat emotional problems [like depression or anxiety], and other people do not. What are your experiences with it?
- Among healthcare services that you attend for depression, which one you most frequently return to?
- When you return to this healthcare service, are you always attended by the same person?
- Have you ever found it difficult to understand what healthcare professional told you about depression? [If so, what happened?]
- Does the health professional who takes care of your depression know with whom you live, where you work, or if you have trouble getting or paying for medications you need?
- Does the health professional who takes care of your depression give you enough time for you to talk about their concerns and their problems with depression?
- Do healthcare professionals that care for your depression, ask about how you are coping with your diabetes?
- What do you do to not feel worse or feel better when you are down or sad?
- What do you do to not feel worse or feel better when you are nervous or worried?
- How satisfied are you with the treatment of depression that you received?
- What do you think, in the treatment of depression, worked well and what did not work very well?
- If you had a choice, what would be the treatment for depression of your choice?

- If you could choose, who would you like to be attended by for your depression, in which health service and at what time?
- Had you received treatment for any emotional problem before a healthcare professional told you for the first time that you had diabetes? [Has the treatment for depression changed since that moment?]
- Do you feel that your preferences/opinions on how to treat depression were taken into account by health professionals? [I wanted to participate more/less or everything is okay?]

(For each of questions 1a, 1b, 1c and 1d, if the answer is positive, you should also ask questions 2a, 2b, 2c, 2d and 2e, before asking the next question in group 1)

- 1a – In any of diabetes consultation, has any health professional told you to see an expert or specialist service for diabetes? [Type of health professional / health service?]
- 1b - In any of the diabetes consultations, did any health professional tell you to see a specialist or specialised service for depression? [Type of health professional / health service?]
- 1c - In any of the depression consultations, did any health professional tell you to consult with a specialist or specialised service for depression? [Type of health professional / health service?]
- 1d - In any of the consultations for depression, did any health professional tell you to consult with a specialist or specialized service for diabetes? [Type of health professional / health service?]
- 2a - Did this health professional / health service helped to arrange this appointment with the specialist or specialised service?
- 2b - Did this health professional / health service know that you made these consultations with this specialist or specialised service?
- 2c - This health professional / health service gave you some information (report or form) for you to take to the expert?
- 2d - This health professional/health service asked to you about what happened during the consultation with the specialist or specialised service?
- 2e - Did this health professional/health service seem interested in knowing what you thought about the care given to you by the specialist or specialised service to which he referred you?

- In the context of our conversation, is there any other topic that is important to you, but you have not had a chance to speak about yet?

Versões em Portugues

Entrevistas com Médicos

- Pergunta de abertura: Você poderia descrever o seu envolvimento, na unidade de saúde em que você trabalha no atendimento de pessoas com diabetes, hipertensão, doenças do coração ou artrite/artrose?
- Existem pessoas que tem duas ou mais dessas doenças crônicas ao mesmo tempo (por exemplo, diabetes e hipertensão ao mesmo tempo). O seu envolvimento no atendimento dessas pessoas tem alguma diferença em comparação com o envolvimento no atendimento das pessoas que tem só uma dessas doenças crônicas?
- Profissionais de saúde contam que percebem que vários pacientes não se sentem à vontade quando contam para eles seus problemas e dificuldades. Essa situação já aconteceu com você? [Na sua experiência, qual paciente fica mais à vontade para contar os problemas e dificuldades e qual fica menos à vontade?]
- Profissionais de saúde contam que várias vezes já tiveram dificuldade para conseguir entender o que os pacientes queriam perguntar ou as explicações deles sobre o que lhes estava acontecendo. Essa situação já aconteceu com você?

  [Isso é comum?]

  [Algumas pessoas acham que idosos/pessoas com pouco estudo podem ter dificuldades em comunicação, e outros não acham isso. Quais são as suas experiências sobre isso?]
- Como você consegue conhecer a situação de vida atual de cada um de seus pacientes, ou seja, por exemplo, onde ele mora, como é a família dele, se ele trabalha ou não, qual é a situação financeira dele, se ele é religioso?

  [Se sim, como você consegue saber essas informações?]

  [Se não, porque você não consegue saber essas informações?]
- Como você consegue conhecer detalhes da história da vida de cada paciente que você acompanha, ou seja, por exemplo, onde ele nasceu como era à família dentro da qual ele cresceu os lugares onde ele morou, o que ele estudou?

  [Se sim, como você consegue saber essas informações?]

  [Se não, porque você não consegue saber essas informações?]
- Como você consegue conhecer o histórico de saúde completo de cada paciente que você acompanha, ou seja, o que aconteceu nos atendimentos que ele já fez em diferentes serviços de saúde e com diferentes profissionais?

[Se sim, como você consegue conhecer essas informações?]

[Se não, por que não consegue?]

- Como você consegue saber se cada paciente que você atende que tem diabetes, hipertensão, doenças do coração ou artrite está recebendo ajuda para alguma dessas doenças crônicas em outro serviço de saúde que não este aqui e aqui onde você trabalha?

  [Se sim, você pode me dar mais detalhes sobre como você consegue saber?]

  [Se não, você pode me dar mais detalhes sobre porque você não consegue saber?]

- Como você consegue saber quais são as medicações que foram receitadas para cada paciente que tem doença crônica que você atende, incluindo aquelas medicações que foram receitadas em serviços de saúde diferentes deste serviço de saúde aqui, em que você trabalha?

  [Se sim, você pode me dar mais detalhes sobre como você consegue saber?]

  [Se não, você pode me dar mais detalhes sobre porque você não consegue saber?]

- Como você consegue saber se os pacientes que você atende estão tomando da maneira certa às medicações que lhes foram receitadas?
- Como você fica sabendo quais são os pacientes que você atende que têm diabetes, hipertensão, doenças do coração ou artrite?

  [Na sua experiência, quais são os melhores métodos de diagnóstico para hipertensão, diabetes, doença do coração, artrite para usar na Atenção Básica?]

- Em situações que você tem de decidir, como você decide qual tratamento é melhor para um paciente com um ou mais dessas DC?

  [Por exemplo, como você decide aconselhar um paciente a tomar remédios específicos, ou fazer algum tipo de atividade física, ou ainda comer determinados tipos de alimentos?]

  [Como é o seu uso dos “Cadernos de Atenção Básica”, do Ministério da Saúde, para ajudá-lo a planejar e decidir qual é o melhor tratamento para os seus pacientes?]

- Quais tratamentos ou formas de ajudar tratar estão oferecidos para hipertensão, diabetes, doença do coração, artrite/artrose na sua unidade de atendimento?
- Desses tratamentos que são oferecidos para essas doenças crônicas neste serviço de saúde, quais deles você acha que ajudam bastante os pacientes e quais deles você acha que não ajudam tanto?

  [Por que você pensa dessa forma?]

  [Você acha que existe alguma combinação de tratamentos que é especialmente boa para ajudar os pacientes com essas doenças crônicas?]

- Se você pudesse escolher, qual seria o tratamento - ou os tratamentos - que você gostaria de oferecer para os pacientes com diabetes, hipertensão, doenças do coração ou artrite/artrose?
- Em sua experiência, pessoas que têm hipertensão, diabetes, doença do coração ou artrite, também tem, ao mesmo tempo, problemas emocionais como depressão ou ansiedade? [Isso é comum?]
- Você acha que hipertensão, diabetes, doença do coração ou artrite podem ajudar a causar depressão e ansiedade? [Se sim, dê mais detalhes sobre como você pensa que as doenças crônicas podem ajudar a causar esses problemas emocionais.]
- Você acha que depressão e ansiedade podem ajudar a causar hipertensão, diabetes, doença do coração, artrite ou artrose? [Se sim, dê mais detalhes sobre como você pensa que esses problemas emocionais podem ajudar a causar doenças crônicas.]
- O que mais você acha que pode ajudar a causar depressão e ansiedade?
- Como é o seu envolvimento no atendimento das pessoas que tem hipertensão/diabetes/doença do coração/artrite ou artrose e também tem depressão e/ou ansiedade ao mesmo tempo? [Você pode dar mais detalhes sobre isso?]
- Você acha que a maneira como cada paciente lida emocionalmente as com essas doenças crônicas - por exemplo, se a hipertensão ou o diabetes deixam o paciente mais triste, nervoso ou, pelo contrário, ele se consegue se manter alegre e tranquilo - pode fazer alguma diferença, para o tratamento dessas doenças crônicas dar mais certo ou dar menos certo? [Fale mais, dê mais detalhes, sobre o que você pensa sobre isso.]
- Você consegue perguntar aos pacientes com essas doenças crônicas como eles se sentem emocionalmente? Por exemplo, você consegue perguntar se eles estão tristes, desanimados, para baixo, ou pelo contrário se estão animados, bem-dispostos, alegres?
  [Se sim, dê mais detalhes sobre como você pergunta sobre isso]
  [De que maneira pacientes respondem pra estar perguntado nesse jeito sobre o estado emocional?]
  [Como você se sente perguntando sobre problemas emocionais?]
  [Se não, dê mais detalhes do porque você não consegue perguntar sobre isso]
- Como você decide quando deve fazer perguntas para um paciente sobre como está o estado emocional dele - ou seja, por exemplo perguntar se ele está se sentindo mais triste, nervoso, com pouca disposição ou, pelo contrário, se está mais alegre, com bastante disposição?
- Alguns profissionais de saúde contam que, na experiência deles, pessoas idosas ou homens tem mais dificuldades para falar de problemas emocionais. Na sua experiência

como profissional deste serviço de atenção básica, isso acontece? [Se sim, dê mais detalhes sobre essa sua experiência]

- Como você lidou(a) com essas dificuldades com conversar sobre problemas emocionais? [Como você pensa que seria uma boa maneira de lidar com essas dificuldades para conversar sobre problemas emocionais cm idosos e homens?]
- Você usa algum método ou estratégia específica para avaliar a gravidade dos sintomas de depressão e ansiedade, em pessoas que você atende neste serviço de saúde que tem, ao mesmo tempo, hipertensão, diabetes, doença do coração, artrite/artrose?

  [Se sim, qual?]

  [Se não, por quê?]

  [Se não, como você fica sabendo quais são os pacientes que você atende que têm depressão e ansiedade?]
- Profissionais de saúde relatam dificuldades para avaliar sintomas de depressão e/ou ansiedade em pessoas com hipertensão, diabetes, doença do coração, artrite/artrose. Como é isso para você? Alguma vez você já encontrou esse tipo de dificuldade no seu trabalho? [Se sim, quais foram os dificuldades?]
- Como você lidou(a) com essas dificuldades com avaliação? [Como você pensa que seria uma boa maneira de lidar com essas dificuldades para avaliar sintomas de depressão e/ou ansiedade em pessoas com doenças crônicas?]
- Como você consegue saber, para cada paciente que você atende, se foram recebidas medicações quem você receitou para tratar depressão e/ou ansiedade?

  [Você pode me dar mais detalhes sobre como você consegue saber (ou sobre porque você não consegue saber)?]

  [Se não, o que você acha sobre isso?]
- Como você consegue saber, para cada paciente que você atende, se em algum outro serviço de saúde foi receitada alguma medicação para tratar depressão e/ou ansiedade?

  [Você pode me dar mais detalhes sobre como você sabe (ou sobre porque você não consegue saber)?]

  [Se não, o que você acha sobre isso?]
- Como você consegue saber se cada paciente está tomando da maneira correta a medicação que lhe foi receitada para tratar ansiedade e/ou depressão?
- Como você consegue saber, para cada paciente que você atende, se ele recebeu alguma ajuda, sem ser medicação, para tratar problemas emocionais como depressão e/ou ansiedade, aqui mesmo neste serviço de saúde? [Você pode me dar mais detalhes sobre como você consegue saber (ou sobre porque você não consegue saber)?]

- Como você consegue saber, para cada paciente que você atende, se ele recebeu alguma ajuda, sem ser medicação, para tratar depressão e/ou ansiedade, em outro serviço de saúde? [Você pode me dar mais detalhes sobre como você consegue saber (ou sobre porque você não consegue saber)]
- Quais formas de ajudar pessoas com depressão e/ou ansidedade são oferecidas no serviço de saúde em que você trabalha?
- Na sua experiência, quais formas de tratamento dão certos e quais formas de tratamento não funcionam bem, para ajudar as pessoas a melhorarem de depressão e/ou ansiedade?
- Se você pudesse escolher, qual seria o tipo de ajuda para tratar depressão e ansiedade, que você gostaria de oferecer para os seus pacientes? [Por quê, você escolheu isso?]
- Na sua experiência, quais formas de ajudar a melhorar de depressão e/ou ansiedade pacientes preferem?

  [Algum paciente prefere mais tomar medicação do que receber algum outro tipo de ajuda para tratar depressão e/ou ansiedade?]

  [Algum paciente prefere receber algum tipo de ajuda que não inclua ter de tomar medicação para tratar depressão e/ou ansiedade?]
- Quando um paciente seu necessita de um encaminhamento para tratamento de doença crônica, você ou alguém da sua equipe de atenção básica consegue conversar com eles sobre os serviços especializados nos quais eles poderiam ser atendidos?

  [Se sim, como você ou alguém da sua equipe consegue fazer essa conversa?]

  [Se não, por quê você ou alguém da sua equipe não conseguem fazer essa conversa?]
- Quando seus pacientes necessitam de um encaminhamento para tratamento de problemas emocionais, você ou alguém da sua equipe de atenção básica consegue conversar com eles sobre os serviços especializados de saúde mental nos quais poderiam ser atendidos?

  [Se sim, como você ou alguém da sua equipe consegue fazer essa conversa?]

  [Se não, por quê você ou alguém da sua equipe não conseguem fazer essa conversa?]
- Você ou outro profissional do serviço de atenção básica em que você trabalha consegue ajudar o paciente a marcar a consulta ou conseguir internação em outros serviços de saúde, se for necessário?

  [Se sim, como você ou alguém da sua equipe consegue ajudar o paciente nisso?]

  [Se não, por quê você ou alguém da sua equipe não conseguem ajudar o paciente nisso?]

- Alguma vez você já percebeu que algum paciente que você atendeu encontrou dificuldades para conseguir atendimento em outros serviços de saúde para tratar hipertensão, diabetes, doença do coração, artrite/artrose, mesmo depois que você ou outro profissional deste serviço de saúde fez algum encaminhamento para esses outros serviços?

  [Se sim, quais as dificuldades que você já percebeu?]

  [Como voce lidou com essas dificuldades?]
- Alguma vez você já percebeu que algum paciente que você atendeu encontrou dificuldades para conseguir atendimento em algum serviço de saúde mental, mesmo depois que você ou outro profissional deste serviço de saúde fez algum encaminhamento para esses outros serviços?

  [Se sim, quais as dificuldades que você já percebeu?]

  [Como voce lidou com essas dificuldades?]
- Quando seus pacientes são encaminhados, você ou alguém deste serviço de atenção básica consegue fornecer para eles informação escrita (um relatório, uma ficha de referência) para levar ao especialista ou serviço especializado?

  [Se sim, como você ou alguém da sua equipe consegue fazer isso?]

  [Se não, por quê você ou alguém da sua equipe não consegue fazer isso?]
- Após seu paciente passar por consulta ou internação em serviço especializado, você ou alguém deste serviço de atenção básica recebe um relatório, uma ficha de contra-referência, desse serviço especializado?

  [Se sim, qual é a qualidade dessa ficha de contra-referência?]

  [Se não, porque você acha que não conseguem receber a ficha de contra-referência?]
- Você ou alguém deste serviço de atenção básica consegue conversar com seu paciente sobre os resultados desta consulta ou internação no serviço especializado depois que ela ocorreu?

  [Se sim, fale mais, dê detalhes, sobre essas conversas]

  [Se não, porque essa conversa com o paciente não acontece?]
- (Pensando em que foi discutindo) como você está sendo sua satisfação com a comunicação com os outros profissionais de saúde, incluindo colegas da sua unidade e de outras unidades de saúde sobre o tratamento dos seus pacientes?

  [O que dá certo?]

  [Quais os aspectos que precisam melhorar?]
- Você tem alguma(s) sugestão(ões) sobre como poderia ser melhorada a comunicação com os outros profissionais de saúde que cuidam de pacientes que você também cuida?

- Você pode recomendar algumas estratégias ou maneiras de integrar os tratamentos para doenças crônicas com os tratamentos para depressão e/ou ansiedade, que dão certo aqui no seu serviço de saúde?
- Você tem alguma(s) sugestão(ões) de como poderia ser melhorada a integração desses tratamentos para esses dois tipos de problemas aqui no seu serviço de saúde? [Por exemplo, ideias sobre treinamento dos profissionais de saúde ou ideias sobre funcionamento de sistema ou equipamento que facilite a comunicação dentro do seu serviço de saúde e/ou com outros serviços de saúde?]
- Pergunta de encerramento: No contexto da nossa conversa, existe algum assunto que é importante para você, mas ainda não teve a chance de falar?

Entrevistas com Pacientes

- Pergunta de abertura: Quando foi a primeira vez que algum medico ou outro profissional da saúde falou pra você que você tinha diabetes [Nota: **OU OUTRA DOENÇA CRÔNICA**, deixe o paciente escolher sobre qual falar e emende outras perguntas de acordo]? [Quem falou isso e no qual serviço da saúde?]

- Em quais serviços da saúde você recebeu atendimento para diabetes [Nota: No caso da pessoa ter passado por muitos serviços ou não se lembrar bem, você pode sugerir para ela tentar lembrar só sobre o último ano]:

a. **Qual o(s) local(is)** de atendimento de **diabetes**?

- ☐ Unidade básica de saúde (posto ou centro de saúde ou unidade de saúde da família)
- ☐ Centro de Especialidades, Policlínica pública ou PAM - Posto de Assistência Médica
- ☐ UPA (Unidade de pronto Atendimento)
- ☐ Outro tipo de Pronto Atendimento Público (24 horas)
- ☐ Pronto-socorro ou emergência de hospital público
- ☐ Hospital público/ambulatório
- ☐ Consultório particular ou Clínica privada
- ☐ Ambulatório ou consultório de empresa ou sindicato
- ☐ Pronto-atendimento ou emergência de hospital privado
- ☐ No domicílio, com médico da equipe de saúde da família
- ☐ No domicílio, com médico particular

b. **Por quais profissionais de saúde** você foi atendido para **diabetes?**

- ☐ médico
- ☐ enfermagem
- ☐ agente comunitário de saúde
- ☐ fisioterapeuta/terapeuta ocupacional
- ☐ educador físico
- ☐ nutricionista
- ☐ farmácia

- [ ] assistente social

c. **Que cuidado** ou formas de ajudar a tratar você recebeu para **diabetes:**

- [ ] Remédios
- [ ] Exames laboratoriais ou de imagem ou exames complementares de diagnóstico (de sangue, de urina, raios-X etc.)
- [ ] Orientação sobre hábitos da vida
- [ ] Outro [Qual?]

- Alguma vez você já encontrou dificuldades para marcar uma consulta ou conseguir tratamento para diabetes? [Quais dificuldades você teve? e.g.: custo, distância para uma unidade de atendimento, horários de funcionamento, tempo de espera para uma consulta em ambulatório especializado ou outros]
- Como você conseguiu lidar com essas dificuldades?
- Algumas pessoas acham que pessoas com cor da pele mais escura ou situação financeira complicada podem ter mais dificuldades de acessar os serviços da saúde para diabetes, e outras pessoas não acham isso. Quais são as suas experiências sobre isso?
- Entre esses locais de atendimento nos quais você foi atendido para diabetes, qual deles é o que você voltou com mais frequência?
- Quando você volta nesse local de atendimento, é o mesmo médico ou enfermeiro que atende seu diabetes todas às vezes?
- Em sua opinião, existe um profissional da saúde ou serviço de saúde que conhece melhor você como pessoa [isto é, conhece pelo menos um pouco sobre a história de toda a sua vida, sobre com quem você mora, sobre sua situação profissional] e conhece melhor a sua história de diabetes?
- Existe um profissional da saúde ou serviço de saúde que você considera que mais responsável pelo seu atendimento para diabetes?
- Alguma vez você já teve dificuldade para entender o que algum profissional de saúde falou sobre o diabetes? [Se sim, que aconteceu?]
- Os profissionais de saúde que cuidam do seu diabetes sabem sobre você como pessoa, além do seu diabetes propriamente dito? Por exemplo, com quem você mora, onde você trabalha, se você tem problemas em obter ou pagar por medicamentos para tratar o diabetes?
- Durante as consultas com os profissionais do serviço de saúde por causa do seu diabetes, eles lhe dão tempo suficiente para falar sobre as suas preocupações ou suas opiniões sobre os seus problemas com o diabetes?

- Os profissionais de saúde que cuidam do seu diabetes perguntam sobre os seus sentimentos? Por exemplo, se você está se sentindo triste, ou nervosa, ou preocupada, ou brava ou com alguma outra emoção? [Você gostaria de falar sobre os seus sentimentos, sobre as suas emoções, com os professionais da saúde que cuidam do seu diabetes?]
- Os profissionais da saúde que cuidam o seu diabetes, te perguntaram o que você gostaria de fazer par melhorar o seu diabetes?

  [Se sim, fale de mais detalhes como isso foi feito.]

  [Se não, você gostaria participar mais em um processo de tomar decisões sobre o jeito de cuidar seu diabetes? Por exemplo, gostaria para um médico discutir com você que forma de ajudar a tratar seu diabetes (com medicação/sem medicação) seria melhor pra você, quais aspectos de sua saúde você gostaria de melhorar (diminuir dor/estar mais capaz de fazer a coisas na casa).]

  [Se fala, "quero simplesmente melhorar", pergunte o que isso significa pra ele/ela]
- Que ajudou e o que não ajudou muito de melhorar diabetes que você recebe ou já recebeu? [Por quê?]
- Se você pudesse escolher, qual seriam as formas de ajudar a tratar o diabetes de sua preferência?
- Se você pudesse escolher, por quem você gostaria de ser atendido para diabetes, em qual serviço da saúde e em quais horários? [Explore atitude da pessoa para participação de vários profissionais da saúde nesse processo. Por exemplo, caso a pessoa fala "gostaria de ser atendido pelo o médico X ". Pergunte: "Porque, só esse médico? O que você acha sobre a ideia da enfermagem atender seu diabetes?"]
- Quando foi a primeira vez que algum medico ou outro profissional da saúde falou pra você que você tinha depressão [Nota: **OU ANSIEDADE**, deixe o paciente escolher sobre qual falar e emende outras perguntas de acordo]? [Explorar: Quem falou isso?]
- Em quais serviços da saúde você recebeu atendimento para depressão?

  [Nota: Caso a pessoa tenha passado por muitos serviços ou não se lembrar bem, você pode sugerir para ela tentar lembrar somente sobre o último ano]:

d. **Qual o(s) local(is)** de atendimento de **depressão?**

- ☐ Unidade básica de saúde (posto ou centro de saúde ou unidade de saúde da família)
- ☐ Centro de Especialidades, Policlínica pública ou PAM - Posto de Assistência Médica

- ☐ UPA (Unidade de pronto Atendimento)
- ☐ CAPS – Centro de Atenção Psicossocial
- ☐ O ambulatório de saúde mental
- ☐ Outro tipo de Pronto Atendimento Público (24 horas)
- ☐ Pronto-socorro ou emergência de hospital público
- ☐ Hospital psiquiátrico
- ☐ Outro hospital público/ambulatório
- ☐ Consultório particular ou Clínica privada
- ☐ Ambulatório ou consultório de empresa ou sindicato
- ☐ Pronto-atendimento ou emergência de hospital privado
- ☐ No domicílio, com médico da equipe de saúde da família
- ☐ No domicílio, com médico particular

e. **Por quais profissionais** de saúde você foi atendido para **depressão?**

- ☐ medico
- ☐ enfermagem
- ☐ agente comunitário de saúde
- ☐ psicólogo
- ☐ psiquiatra
- ☐ terapeuta ocupacional
- ☐ assistente social
- ☐ Outros [Qual?]

f. **Que cuidado**/tratamento você recebeu para **depressão**:

- ☐ Remédios
- ☐ Psicoterapia [por exemplo, conversar sobre o problema com psicólogo]
- ☐ Orientação sobre hábitos da vida
- ☐ Outros [Qual?]

- Você se sente confortável de conversar sobre problemas emocionais com os outros?
- Com quem você costuma conversar sobre os seus problemas emocionais? [Exemplos: família/amigos /médico de família/psiquiatra/outros profissionais de saúde/outros?]
- Algumas pessoas acham que o diabetes pode ajudar a causar depressão ou tem impacto na depressão. O que você acha?
- No contrário, algumas pessoas acham que depressão pode ajudar a causar diabetes ou tem impacto na depressão. O que você acha?
- O que mais você acha que pode ajudar a gerar/causar do sua depressão?
- Desde que você ficou sabendo que tem diabetes, você encontrou dificuldades para marcar uma consulta ou conseguir tratamento para depressão? [Se sim, quais dificuldades você teve: custo, distância para uma unidade de atendimento, horários de funcionamento, tempo de espera para uma consulta com ao atendimento especializado ou outros]
- Como você conseguiu lidar com essas dificuldades?
- Algumas pessoas acham que pessoas com cor da pele mais escura ou situação financeira complicada podem ter mais dificuldades para conseguir ajuda nos serviços

de saúde para tratar problemas emocionais [como depressão ou ansiedade], e outras pessoas não acham isso. Quais são suas experiências sobre isso?

- Entre esses locais de atendimento pra depressão, qual deles é o que você voltou com mais frequência?
- Quando você volta para o atendimento para a depressão nesse serviço, é o mesmo profissional da saúde que faz o atendimento para a sua depressão todas às vezes?
- Alguma vez você já encontrou dificuldades de entender o que os profissionais da saúde falaram para você sobre depressão? [Se sim, que aconteceu?]
- O profissional da saúde que cuida da sua depressão sabe com quem você mora, onde você trabalha, ou se você tem problemas em obter ou pagar por medicamentos que você precisa?
- O profissional da saúde que cuida da sua depressão lhe dá tempo suficiente para você falar sobre as suas preocupações ou seus problemas com depressão?
- Os profissionais de saúde que cuidam da sua depressão, perguntam sobre como você está lidando com o diabetes?

(Para cada uma das perguntas 1a, 1b, 1c e 1d, no caso da resposta ser positiva, você deve fazer também as perguntas 2a, 2b, 2c,2d e 2e, antes de fazer a próxima pergunta do grupo 1)

- 1a - Em algum dos atendimentos para diabetes, algum profissional de saúde falou para você consultar com um especialista ou serviço especializado para diabetes? [Tipo de profissional da saúde/ serviço de saúde?]

- 1b - Em algum dos atendimentos para diabetes, algum profissional de saúde falou para você consultar com um especialista ou serviço especializado para depressão? [Tipo de profissional da saúde/serviço de saúde?]

- 1c - Em algum dos atendimentos para depressão, algum profissional de saúde falou para você consultar com um especialista ou serviço especializado para depressão? [Tipo de profissional da saúde/ serviço de saúde?]

- 1d - Em algum dos atendimentos para depressão, algum profissional de saúde falou para você consultar com um especialista ou serviço especializado para diabetes? [Tipo de profissional da saúde/serviço de saúde?]

- 2a - Esse profissional da saúde/serviço da saúde ajudou marcar essa consulta com o especialista ou serviço especializado?

- 2b - Esse profissional da saúde/serviço da saúde soube que você fez essas consultas com esse especialista ou serviço especializado?

- 2c - Esse profissional da saúde/serviço da saúde te deu alguma informação (relatório ou ficha) para você levar para o especialista?
- 2d - Esse profissional da saúde/serviço da saúde perguntou para você sobre o que aconteceu durante a consulta com o especialista ou serviço especializado?
- 2e - Esse profissional da saúde/serviço da saúde pareceu interessado em saber o que você achou sobre o atendimento que lhe foi dado pelo especialista ou serviço especializado para o qual ele encaminhou você?
- <u>Pergunta de encerramento:</u> No contexto da nossa conversa, existe algum assunto que é importante para você, mas ainda não teve a chance de falar?
