# Supplementary material for: Mental-physical multimorbidity treatment adherence challenges in Brazilian primary care: A qualitative study with patients and their healthcare providers
Source: PLoS One. 2021 May 13;16(5):e0251320. doi: 10.1371/journal.pone.0251320 (PMC8118469; doi:10.1371/journal.pone.0251320)
Supplement: S2 Appendix — (PDF) [file pone.0251320.s002.pdf]

S2 Appendix

The thematic tree

1) Variability and accessibility of treatment options available through PHC
- (1.1) Variability of usual care options available through primary care
- (1.2) Importance of collaborative care
- (1.3) Coping strategies used by patients to manage psychological aspects of disease

2) Importance of coming to terms with a disease for treatment initiation
- (2.1) Importance of illness acceptance
- (2.2) Impact of physical symptoms on treatment acceptance

3) Importance of person-centred communication for treatment initiation and maintenance
- (3.1) Individualized interactions tailored to a patient
- (3.2) Comprehensive and understandable information

4) Information sources about received medication
- (4.1) Medical records systems use
- (4.2) Relaying on patients' self-reports
- (4.3) Presenting prescriptions and medications
- (4.4) Home visits to review prescriptions and medications

5) Monitoring medication adherence
- (5.1) Routine monitoring of health parameters
- (5.2) Looking out for signs of medication shortage or over-accumulation
- (5.3) Primary care pharmacy computer records use
- (5.4) Seeking information from family members
- (5.5) Home visits to monitor medication adherence

6) Taking medicines unsafely
- (6.1) Not taking medication on time
- (6.2) Using the wrong amount
- (6.3) Failing to check with doctor before stopping
- (6.4) Failing to report adverse effects
- (6.5) Suspected medication sharing

7) Perceived reasons for medication non-adherence
- (7.1) Difficulties with access to medication
- (7.2) Change of or multiple prescribers
- (7.3) Impact of co-existing mental health disorder
- (7.4) Difficulties with following instructions how to take medication
- (7.5) Beliefs about consequences of a medication use
- (7.6) Religious beliefs interfering with medication use

(7.7) Difficulties with incorporating medication in daily routine/lifestyle

8) Most challenging health behavior change goals

(8.1) Lifestyle changes

(8.1.1) Lack of commitment to making lifestyle changes
(8.1.2) Lack of understanding of the importance of lifestyle change
(8.1.3) Difficulties with changing and creating new habits
(8.1.4) Emotional dimensions of unhealthy habits
(8.1.5) Social influences on lifestyle choices
(8.1.6) Limiting environmental context and resources

(8.2) Depression and anxiety drugs abuse and misuse

(8.2.1) Incorrect use of antidepressants and anxiolytics
(8.2.2) Widespread use of benzodiazepines to manage sleep problems
(8.2.3) Antidepressants and anxiolytics dependency
(8.2.4) Perceived reasons for anxiety drugs abuse and misuse

(8.3) Insulin injections

(8.3.1) Reluctance to use insulin therapy
(8.3.2) Efforts to overcome patients' resistance to insulin therapy
(8.3.3) Endocrinologist referrals when efforts to monitor insulin therapy failed

9) Main motives for initiation or maintenance of treatment

(9.1) Motivation to maintain treatment
(9.2) Behavioral regulation of treatment adherence

10) Methods deployed to improve treatment adherence

(10.1) Social network involvement
(10.2) Extra measures for patients in need
(10.3) Strategies to assist with organization of medication
(10.4) Enabling access to prescription medication

Exemplary quotes

List of Abreviations

English		Portuguese
**BHU**	Basic Health Unit	Unidades Básicas de Saúde
**CHW**	community health worker	agente comunitário de saúde
**FHS**	Family Health Strategy	Estratégia Saúde da Família
**MHCC**	Mental Health Community Centre	Centro de Atenção Psicossocial
**MMHS**	manager of mental health service	coordenador(a) do serviço de saúde mental
**NA**	nursing assistant	auxiliar de enfermagem
**PCMHP**	primary care mental health professional	profissional de saúde mental da atenção primária à saúde
**PCP**	primary care patient	paciente da atenção primária à saúde
**PHY**	physician	medico(a)
**RN**	registered nurse - not a manager	enfermeiro(a) não coordenador(a)
**RN/M**	registered nurse – primary health care unit manager	enfermeiro(a) coordenador(a)
**TSSP**	Team of Specialists Supporting a Primary Health Care unit(s)	Núcleo de Apoio à Saúde da Família

Notes:

For improved visibility, PCP quotes are written if blue font.

Transcription conventions: (..) – silence; less than one second; he says. – emphasis; [notes, comments]; […] - part of the text ommited to reduce the length (some of the markings were removed to improve readability. For full list please refer to: Bailey et al. (2008): doi:10.1093/fampra/cmn003)

1) Variability and accessibility of treatment options available through PHC

(1.1) Variability of usual care options available through primary care

''I think that the treatment [for chronic disease] tend to be, here at a Basic Health Unit, more of medication and guidance. We don't have more to offer, this is usual.'' (PHY 9 - BHU, female)

''When I make a diagnosis for this patient, I always start the treatment with video guidance on habits, changing eating habits, I encourage physical activity. This kind of stuff; and I ask this patient to return, make a return with less time then, in this next consultation we talk and see how he is doing. [...] There's a nutritionist here. But there is no other treatment method of this type [multidisciplinary], which I know is not offered here.' (PHY 1- BHU, male)

''Here we have the normal [FHS] consultation, depending on the need we have a home visit with the nurse, with the doctor; if the patient is bedridden, homebound, the girls [nursing staff], the nursing assistant go home to do this pressure and diabetes control. If a patient can do it, he/she comes to the clinic, there is the medication, we have a pharmacy here, which is one of the few stations that have a pharmacy, here there is, or the person goes to the center too. And we usually have medication, in case people come with a crisis of high blood pressure, we measure it on the spot, already on the spot, that not all the primary care units have, but this is because our nurse takes upon herself the task to request it, and have these medications in case of an emergency, which sometimes happen. Also, we used to have a group, our group of elderly diabetic and

hypertensive people, for which we did walking, memory exercises.'' (CHW 5 – FHS, female)

''We offer in addition to the usual that I already spoke about - the medical part, like most of the time drug treatment, but also guidance focused on the patient's health, in general to improve the quality of life - we usually do activities, like there are walking days, when the [community health] workers go out with patience and recruit the people to walk, to do physical activity, food guidance, we usually give a lecture every month to try to guide the patients' diet.'' (PHY 3 – FHS, male)

''I won't be able to say all the names, but there is the hypertension group, the diabetic group. In the orthopaedic part there is not. [...] I will not remember exactly, but I know that girls [nursing staff] do it well and I try to implement it, they do it before the consultation: they present some boards, talk about the disease. We encourage them, it's very interesting''. (PHY 4 – mixed FHS & BHU, male)

''We do lectures about diabetes, high blood pressure, we have a colleague of ours, trained in physical education, we are doing a stretching group in the morning, then we have a chat, we focus a lot on this part - food, so we are always develop a lecture with them. We do [group] walking from Monday to Wednesday and on Fridays'' (CHW 1 – FHS, female)

''The nutrition and health group has more public [interest], but we have other [groups] that also show good results - in the walking group that we have here, are more than 20 people'' (CHW 2 - FHS, female)

''As we opened a pool next to the unit, we are trying to set up a water aerobics group for the elderly and this is already working.'' (PHY 7 – FHS, male)

''We have a service nearby, which is called, what do you call the center? Ah, yes - ''Holistic Center of Light''. This service is nearby. There are therapists, volunteers and there it is: reiki, tai chi, yoga, acupuncture, all for free. Acupuncture with a doctor, family doctor, even this service. It's all free. There are groups of, oh! I don't remember the names very well, but family therapies, right? I don't have the specific name for that. There are several people that we refer to them. They are from the neighbourhood [..]. I've had the opportunity to go that place, [it's] wonderful. It has crystals, it has nothing to do with religion. The owner is a woman. Therapists are all volunteers and it's very cool. So there's a patient who goes there and is very good. [It's good for] especially those with chronic diseases and those who want medicine for insomnia, because they don't sleep and stuff. She [the owner] came here once, we called [patients] to give a lecture, one of the therapists, he came and said: "Yeah, you keep bugging the doctors, to give you a tranquilizer, to give you medicine and that's not right, you already take so much, people, let's go, let's move on to other things". So, this, this is something more alternative, right? I think this complementary medicine is very useful. They came, we've done it several [times].'' (PHY 11 – BHU, female)

''We have here [at the unit] a social worker, family doctor, speech therapist, psychologist, nutritionist, who is currently on maternity leave. Then, we have the unit's physiotherapist too, okay. I think it's just (..) The community workers. That is (.) There is a practice nurse and an assistant, right? [...] there's the nursing assistant. There is auriculotherapy too. Which is the one that puts seeds on people's ears, but it's another professional who makes it and even uses this room that we're in. It works, even for a person who uses medication, can put [seeds] on the diabetes areas, hypertension area, anxiety area. (CHW 8 – FHS with TSSP, male)

''We don't have any specific treatments for anxiety and depressive disorder. But the demand is very high. There should be. But we don't have it. But for me it's a matter of educating my team, there should be a training on how to deal with this type of [problem] [...] Because it usually ends up not being recognised, not making depression management here easier''. (RN/M 1- FHS, female)

''We have only a clinician, so the only service that is offered [for mental health problems] here is referral, and so they [patients] have to wait.'' (CHW 2 – FHS, female)

"Usually when the patient requests this type of medication [psychotropic] I question 'why' and 'how', most of the time the medications are taken in the wrong way. So, most of the time I end up not renewing the prescription and asking a patient to look for specialised care to treat their psychiatric problem" (PHY 1- BHU, male)

''I don't have too many things for this part [depression and anxiety] and it's more about talking that I do, the analyst part, here we mange to convers, here we try to clarify the problem, at this unit, here is more with two [community health] workers visiting, or the first one [CHW] she has this great approach, you haven't' seen her here today, but you've met her before. [Name] she has a great approach. She calls a patient inside the room and speaks a lot, to know what the problem is, it's more integral [approach].'' (PHY 3 – FHS, male)

‘’[I manage] both new cases, as well as a continuation [of psychiatric medication]. If it happens that I have a complaint and I make the diagnosis of depression or anxiety and I often start with medication’’. (PHY 10 – FHS, male)

‘’I try to treat people here with us [at the PHC unit]. I try to start with medication, to accompany a person more closely, I try to give that continuity. I try to refer for psychotherapy, I speak to a social worker, I try to get to know the patient better.’’ (PHY 4 - mixed FHS & BHU, male)

‘’[When] I see that it [depression] is poorly controlled, I write a reference letter, direct to the MHCC, which is where he/she is taken care of by the psychiatrist. There the demand for them to be renewing [prescriptions] is high too, so they send [patients] back there [to primary are units], to their area that is renewing, we are strictly for renewing [psychotropic prescriptions] for them’’ (PHY 3 – FHS, male)

‘’There is a psychologist [visiting] twice a week, a nutritionist too, a social worker too, for some types of care outside the [primary care] unit, there is a unit outside that is also in the public [health system] network, with neurologist, orthopaedist, psychiatrist; and sometimes the psychiatrist consult in the MHCC, and there they are referred [by the psychiatrist], but we remain attentive if he/she [a patient] is not being looked after here at the unit, even so, we keep up to date with the information.’’ (CHW 4 - mixed FHS & BHU, female)

‘’We have the advantage of receiving the fifth year students of psychology here, and we end up having a group, which we call the Community Mental Health Group, which is a space for people to talk about life, it is not about mental health in particular, but it is to share frustrations, fears, joys, it is a therapeutic space and we end up having, this year we already have had two or three [encounters], they organise it. So in this place, which is a great help, I think and the population attend this group.’’ (PHY 10 – FHS, male)

‘’In a psychiatric consultation that takes place [here] every Tuesday, there is a consultation that involves all the doctors, nurse, the doctors involved in this patient’s care.’’ (CHW 12 – FHS, female)

‘’At the beginning, I always try to offer the psychologist. They want it because on the [public] network we don't make referral to a psychologist; we give them the phone number they call and schedule [an appointment]. So I ask, right? If the patient says that he has no interest [in seeing a psychologist], that he does not want to, I do not even waste time offering it, providing the number, for example. But whoever has it, expresses interest, I already do it, we will start treating it. I already ask to come back in a month, so we can see how it is. Try to fit, come fit in the beginning of the month on my calendar, but I want to see, right? […] we don't send to MHCC psychologist, do you understand? I only send it to MHCC when I need a psychiatrist. Do you understand? There on the network, there are psychology services [university name], [university name], which are also for free. Then they pass the number and then the person will call and get there’’ (PHY 11 – BHU, female)

‘’In the primary care we have already done that, there was a support group for women. (..) They are women with a psychiatric problem, but we did not make it so clear to them. We, we, we formed this group, yes, we selected patients who already had some type of [psychiatric] problem or involvement in mental health [services]: or mild cases of depression or anxiety. Not that they didn't know [they had that problem], they knew. They knew. They were aware that that group was made up of women who, women who did some follow-up [with health services] but were not focused [on a particular problem] - I think my I’m not explaining myself well (..) – it [focus] was not directed at mental health. It was a group for women that had issues, but not in mental health, even though they all did. And it was a group that lasted long time (..) about four months, with a beginning, middle and end. Ending was very interesting; we went to a park in [city name]. It is a park and it is called [name] and we finished it there.’’ (PCMHP 1 – FHS with TSSP, female)

‘’The professional, family doctor or clinician who is there (..) in the FHS with the support of TSSP, he/she is feeling a little more comfortable to provide this [mental health] service. Because, its’ what normally TSSP guideline recommends – [primary care-based] care (..) for mild to moderate [cases]. Serious cases can be seen by the individual psychiatrist.’’ (PCMHP 1 - FHS with TSSP, female)

(1.2) Importance of collaborative care

‘’I think it is a question of guidance with mental health professionals or the question of training people to deal with this type of issue. In Ribeirão I had this experience and the TSSP professional was a great partner of ours. There was a lot of connection, the patients we referred, there was an exchange. But here we are the discovery stage, let alone TSSP. I don't see any possibility in five or ten years’’ (RN/M 1 – FHS, female)

‘’I believe that this HIPERDIA program showed good results, patients attend it. (..) They come to the appointment, you don't have to come in the morning to make an appointment, you don't have to get up early, the appointment is scheduled every six months, every five months with the nurse. The nurse takes the exams and a patient comes with them, he comes every five months, he passed the consultation today, the exam is

all right, everything is good, everything is right, his exam, we already have him scheduled in five months. He comes back with five months and a prescription is valid for six months. He comes back with five months. With the months he came back, the nurse has already arranged his exams so he can return with twenty days so he can get the prescription with the exams ready. That he takes every six months that he is in control of his progress. It's a very good program. In this program is offered, there is a girl [physical educator] who went to explain to them theire exercise for them, the importance of exercise, all that part. There is a nutritionist who guides the food, you don't even have to be hypertensive and diabetic'' (NA 4 – mixed FHS & BHU, female)

''The ones that help the patients the most are the groups and individualised care with the multidisciplinary team. When the patient passes and receives only the guidance of a member of the nursing team, he does not take on board much. When he is in a group or in individualised care, he has the ability to interpret his problem and share the problems with his peer group and they find a common ground. And in individualised care he can better express his difficulties and what he can do to improve'' (NA 2 - mixed FHS & BHU, female)

''We could offer better mental care] if we had access to a TSSP, which has a psychologist, which provides access to psychology.'' (RN/M 1 – FHS, female)

''Oh! I don't know, but I would really like healthcare professionals to help me, not necessarily the TSSP, the TSSP would be ideal, but if there was a nutritionist or something that could look, the diabetic patient, the patient made the diagnosis, let's talk about diet, sit there with her, she will teach you about a diabetes diet. [Like this:] ''come on, let's change lifestyle, let's talk about physical activity, so the fitness coach sits there and does a series of physical activities for the person.'' (PHY 10 – FHS, male)

''If there was a follow-up with a nutritionist, you know, a physiotherapist like this kind of specialties they need to search from time to time, that they could have easier access, but we don't have it.'' (CHW 2 – FHS, female)

''Multidisciplinary work is always more effective. Yes! It would be great [to have supervision also from a mental health professional]. Would be great!'' (PHY 9 – BHU, male)

“There [in the PHC unit] I would provide a physical education teacher. A professional there.” (PCP 8 – mixed FHS & BHU, female)

“I wish there [in the PHC unit] was a psychologist who could assist me. Yes, to atend me.” (PCP 12 – FHS, female)

“It would be good, it would be very good [to have here - in the PHC unit] nutritionist, psychologist.” (PCP 2 – FHS, female)

“I wish we had [here – in the PHC unit] a cardio [cardiologist], right? So you can see (..) And I don't know which doctor treats arthritis but if I had one too, it would be good.” (PCP 4 - FHS, female)

“If you brought the psychiatrist here [to the PHC unit], it'd be better, right.” (PCP 9 – FHS, female)

“A psychologist I would even like to see, because I also do not have one, one, one here. Is there even a psychologist that [depression]? […] Perhaps, (..) he/she could give me hope, a change in the thing. (..) But I never had that opportunity.” (PCP 10 – BHU, male)

(1.3) Coping strategies used by patients to manage psychological aspects of disease

“I talk to God a lot. So, I tell God my problems. So if you have a person you can talk to, right? It's good.“ (PCP 4 – FHS, female)

“Cry a lot. I cry, I get better. Today I'm sad, because of a fact that happened, but I will hold on. And it's like that. I cry a lot. Crying is good.” (PCP 12 – FHS, female)

“[…] so everything that happens to me is with her [neighbour] that I talk to, right? And she too, you know? The two of us is an outburst to each other, like that, so I think that's my salvation a little bit, right?” (PCP 6 – FHS, female)

“We [PCP 3 and her son] talk about everything, about how the situation is at our house, how it is, there are doctors talking about everything, it helps, it helps a lot.” (PCP 3 – FHS, female)

“I try to pretend I'm not worried, disguise as much as I can, I really like to read, I pick up a book I'm going to read. [...] I can't sleep, I pick up a book and I'd read, I'd read until I fall asleep. " (PCP 2 - FHS, female)

“Yeah, I make my rugs, sometimes I play a game, sometimes I'm alone and I play a game you know? Then I am distracted [...] My granddaughter has a cell phone and has a lot of games they have puzzles, lots of things there (..) as long as I am distracted [it's ok].” (PCP 15 – BHU, female)

2) Importance of coming to terms with a disease for treatment initiation

(2.1) Importance of illness acceptance

‘’Well, if the person doesn’t accept that problem, right? Or she/he has a lot of difficulty with the treatment, right? This generates anxiety, an internal conflict and it can interfere, right? [interfere] With the treatment results.’’ (RN/M 9 – BHU, male)

“There is a relationship, as soon as you accept [a disorder] you take better care, when you don't accept you take [medication] it any which way, take it at whatever hour, at any time. If you didn't take the medicine at the right time, so it does interfere [with adherence], when you accept you believe that you will have an improvement, that you will get better.“ (CHW 6 – FHS, female)

“I was shaken out [of my apathy] by my husband, he said: “It is not like that, you cannot do this, you cannot throw yourself on a bed because you have diabetes, diabetes will not kill you, what will kill you is your dismotivation, because you are too discouraged, it’s not like that .” He said: "Go out and talk to the doctor, she will explain what you have to do". That's where I went at that time and started to treat” (PCP 2 – FHS, female)

(2.2) Impact of physical symptoms on treatment acceptance

‘’Diabetics are the most difficult to approach. Because it is a patient who is difficult to put on a diet, follow treatment, they are resistant to medication, side effect, they do not understand the importance of the disease, especially [those] with both hypertension and diabetes, because they have no symptoms, right, most of the time.’’ (PHY 4 – mixed FHS & BHU, male)

"It happened that a patient of mine, he was treating hypertension and diabetes. “Look you have to treat it” and it was always the same story: ‘’I am not going to treat’’. He did not treat anything in the right way. Then he suffered a heart attack, got scared, because he was [grateful for] being still alive, it gave that shock, [he] came back and started wanting to treat everything right." (PHY 3 – FHS, male)

“One day, I was very ill, and the doctor was the first thing he said: “has anyone done a blood test to see if this girl does not have diabetes? [...] Before you put the serum in [saline solution], said, let's do it.” It was almost four hundred (..) But I didn't believe it. I didn't believe it because I didn't feel anything, I didn't feel much...lots of urine and [drinking] water, all that. […] It reached a limit, four hundred and so much [blood glucose]. Then I stayed in the ICU, monitored, everything. It was a horrible experience (..) I said to myself: “I'm dying” (..) So, as you are a layman on the subject, you say: “man, which one is going on, why am I here? What did I do that was so wrong? […] [We] start to understand that you really have a serious health problem, so that you have to take action. And this is what I took (..) I became aware. So I started the treatment." (PCP 17 – FHS, female)

“It was that he [a doctor] discovered that he [the patient’a husband] had a tumor and I felt bad, very bad. Close to him, at the doctor, at the time he found out, when the doctor said he had this disease, back then no - I didn't feel it [bad]. I got ‘knocked dow’ but I held it, but after he had a surgery, everything [post-operation] and [at thta time] I felt bad, I felt my heart racing, you know? A lot. Then I: “people, this is not right.” Then I went to "measure" the pressure, it was high, 14x10, from then on I started taking medicine. (PCP 12 – FHS, female)

3) Importance of person-centred communication for treatment initation and mantanance

(3.1) Individualised interactions tailored to a patient

"I always use [questions like]: “Oh, what are we going to do? Are we going to start this medicine?”. Then they [patients] sometimes also say: “What are we going to do now? And what are we going to take?”. So it’s interesting to see him/her saying this to me, you know. What are we ‘’going to take’’, as if it was together because when I speak it is like taking it together, and when they say it, then I say: "Wow, so it seems that you are feeling trust" (PHY 5 – FHS with TSSP, female)

“So it is, I think, the only way that patients have to find out how we are dealing with them. If I'm only interested in his illness, his hypertension, or if I'm interested in Mr John, who has hypertension. So, the ideal would be

that, if we started with the patient [with a question] “how are you doing?”, and not just thinking about the parameters of the disease. And I think that when he can capture that, that we have an interest in that, when he can do that, we create a greater bond. And then it is easier for the patient to adhere to the treatment because with this bond the day he comes back here he will think twice before not doing what was proposed, what was agreed. It becomes a combination, a deal we make between the doctor and the patient. Then when he comes back, he already thinks: "I will not do the wrong thing because I will have to tell the doctor and she will not like it". The bond remains. So when we can do this, when you can catch the patient and show that you are interested in him and not in the disease, I see, then the treatment works much better. “(PHY 9 – BHU, female)

“I think for the patient to adhere to the treatment that you are proposing to him/her, he/she has to trust what you are saying. So, listening to the patient is very important in this case because he/she will realise that you are interested in what he/she has, you are not going to just give a medicine, you are interested in helping him in this other part. It helps a lot in patient's adherence to treatment, him/her accepting your suggestions in relation to this [what he has].” (PHY 6 – mixed FHS & BHU, male)

“He [a PHC physician] didn't even look at your face, you were going to talk, and he would cut the conversation short, you know? You were going to explain, and he wouldn’t hear it, cut it, you know? [End of] the conversation, he was already giving a prescription.” (PCP 15 – BHU, female)

“Because she is a family doctor, she is a doctor who is really interested in people's wellbeing [...] She asks what happened, if anything happened, if I fought with someone, or if someone fought with me, she would ask this. It is the doctor who makes the difference, she is attentive, she wants to know everything we have to say, she has time to listen, to talk, to explain, I think this is it.” (PCP 2 – FHS, female)

“Having this [kind of] doctor to attend you, right? What else do you want? In addition to creating a bond with him [...] it becomes so, trustworthy. You come here and make effort, and he's there. Then he would say: “Look, so-and-so, how are you?” I mean, he knows your name, right? He knows who you are. [He asks:] "Is everything ok? Mom, husband”, and so on.” (PCP 17 – FHS, female)

“With her [a specific FHS doctor] I could do it [feel free to talk]. Great, I talked a lot, wow! She paid attention. I think this is what you need, sometimes at a post [a FHS unit]. A person who listens, a person that you can put out [resurface] your problems, right? That sometimes helps you.” (PCP 12 - FHS, female)

“[it’s important for] the doctor to exchange a little more ideas about what the person is going through at that moment, in that period of his/her life, to exchange a little more ideas with that person so that the person can loosen up and express themselves better. And to have a little more freedom and time ... To dialg with the doctor, not to simply get there as it is today: with a queue, in this rush and that "Just watch your pressure and here, take this medicine here". So, a better service, in a secluded room [quiet, private, and undisturbed], with a longer consultation period.” (PCP 13 – mixed FHS & BHU, male)

“I think I could share a little bit of my struggle. Share a little with someone, tell, right? Talk (..) about my day-to-day. I think it would also help, right? We can talk. Ah, with more attention from the professional, right? Because since you go there every six months (..) Yeah, better attention, right? To find out how these six months are going, how I have dealt with depression, right? If things inside my house have improved, right? If I'm calmer.” (PCP 4 – FHS, female)

“He [a PHC physician] didn't even look at your face, you were going to talk, and he would cut the conversation short, you know? You were going to explain, and he wouldn’t hear it, cut it, you know? [End of] the conversation, he was already giving a prescription.” (PCP 15 – BHU, female)

(3.2) Comprehensive and understandable information

"So, we try to make drawings, use every possible resource and available strategy, to try to make the patient understand what he/she has, because if he/she doesn't understand, he/she won't adhere, if he/she not doesn’t understand what each medicine is for, why he/she has to use that, why he/she cannot eat sugar, he will not treat it. " (RN 5 – university FHS, female)

"Then the importance of making a longer consultation, a little bit better-made one, right (..) At least fifteen minutes, right, to also talk about other things, to guide the patient well" (PHY 6 – mixed FHS & BHU, male)

‘’For that reason [more information] the patient with chronic diseases, diabetes, ends up staying for twenty minutes, thirty” (PHY 11 – BHU, female)

“I felt good, because he gave me a correct explanation, I was in doubt and he answered my query. I remembered his guidance, I said: "I'm going to start walking to see if I get better", I said to him "Doctor, does a treadmill works?". He said ‘’No, only when it’s raining, when it’s not raining you go for a walk, you meet people, when you meet a person you say ‘’Hi how are you?’’, then you encounter with other’s ‘’how are you’’, he said that what helps the walk, also helps your psychological.” (PCP 3 – FHS, female)

"[A] piece of paper. In writing [...] he [a doctor] explained that I had to change the diet a lot. Eat more cooked things, less fried food, certain types of fruit, do not eat much, eat in between. Oh, and not eating too much greasy stuff [...] And also not using alcohol, which I never did anyway, I never drunk. So I think this helped a little too.” (PCP 13 – mixed FHS & BHU, male)

‘’[The] only thing the doctor said to me: “you have to lose weight, you have pressure like that because you are fat, right?”. I was weighing as much as a hundred and twenty kilos at that time. Even I agree, I was very, you know, above. But he said to me: “you have to stop eating everything” (..) I am pre-diabetic. Pre-diabetic, that's why I’m telling you this: information about obesity is lacking, right. If there were, like, in all health units, set up a group to treat, raise awareness: fat is a disease, right’’ (PCP 8 – mixed FHS & BHU, female)

“He [a private doctor] did not explain anything, right? [The one] at the primary care neither. He said that “diabetes is dangerous, you have to know how to control it because it cannot rise or fall too much, because everyone goes into a coma’’. That's what he said to me.” (PCP 14 – BHU, female)

“He [the BHU doctor] does not specify how to do physical activities and how to do food reeducation [reduce calorie intake]. He just tells me when I'm overweight, to look for a nutritionist, some professional in the area, to do food reeducation, to lose weight, to help with pressure.” (PCP 13 – mixed FHS & BHU, male)

4) Information sources about current pharmacological treatment

(4.1) Medical records systems use

‘’And then I find out, asking, most of the time or in the same way. I will even check in Hygia [an electronic health record system] - I go to ‘medications received’ [at a MHCC] and I see, but it depends a lot on the person’s information. It happens not having a letter of reference, nor access to the electronic health records.’’ (PHY 10 – FHS, male)

“Many of the patients come here and do not bring the prescription, and sometimes there is a colleague who does not annotate, it doesn’t get registered, nowadays it is easier because if you prescribed something in the system, the system itself attach it. But if the professional, even here at the primary care unit, makes it by hand [written recipe], by hand, and does not put it in [the system], or if he makes a recipe that does not use the system's recipe, then sometimes I don't know. ” (PHY 5 – FHS with TSSP, female)

“The problem is that sometimes there are several prescriptions that are not from SUS [public healthcare system], right? So they are not in the system. So in the ‘pharmacy icon’ I can see what the patient got from SUS. But in, in […] There are some drugs that are not, so I don't know.” (PHY 11 – BHU, female)

(4.2) Relaying on patients’ self-reports

“We ask what medications they are taking and they report to us which medications, there are some [patients] who sometimes forget because they take too much medication, but at one home visit or another, we end up completing this [information] there.”(CHW 7 – FHS, female)

“I usually find out through the patient, by asking the patient: “Did you go there [another healthcare service]? What has been done [there]? Did you get better? You didn’t get better?” Through the patient, it's not through the doctor here, in the system.” (PHY 6 – mixed FHS & BHU, male)

“I can only know if the patient himself/herself tells me. But I don't have access to any place where I could have this information [about treatment prescribed by other doctors]. Only if the patient says that he passes other units [healthcare units]” (PHY 1 – BHU, male)

“Elderly people have more problems remembering to bring medicine, of course they have memory problems. Now, when they change, I put prescription medication, dose, all in the medical records. A medical record is a sheet of the patient's life, visits that he had.” (PHY 2 - FHS, female)

“No, I can't get that information [about medication] either. For one, because of the [a lack of electronic] medical records. Another reason being, let's say, due to the problem of [limited] consultation time, a length of stay in a

specific BHU, we [BHU physicians] end up changing a lot, so there's no time to have this contact, this knowledge of patients." (PHY 1- BHU, male)

"[in four years] (..) I've passed through many doctors (..) first I think it was doctor [name 1], then was that one [name 2] (..) I think he was first, yes, it was doctor [name 1], then doctor [name 2]. [Name 2] attended me only once (..) Then another doctor came. I don't remember who she was, Dr. [name 3] or [name 4], something like that (..) Then finally, now another doctor (..) So when I go [to see a doctor], it has to be, it has to start all over again." (PCP 13 – mixed FHS & BHU, male)

"I would like it to be the same doctor, right? Isn't it true? Because one needs to tell the other, because you don't know well, you are seeing the exams there, but you don't know, right? Each one thinks something [different], I would like to always be the same doctor (..) even my daughter who accompanies me, she says: "Mother, every time a [different] doctor, every time!?" (PCP 15 – BHU, female)

"This business of changing doctors today, changing doctors tomorrow. In the end, you are in troubles with everyone. Isn't that so? Because you are going today, then you express everything to a doctor. I even went there, talked to Dr. [name 1] and explained everything to him, okay. He gave me what [medication] was to be taken, everything. Then I went back there, it was no longer Dr. [name 1], the other doctor was there already. Now is no longer that doctor, now is a she doctor. Are you understanding how it works? So you can never deal with anyone, because then it becomes that snowball, neither one nor the other. So look, I have seen so many doctors, that I don't even know how to explain. It has been many doctors already (PCP 12 – FHS, female)

"I have, I have to explain. I explain. I explain, I think have already explained for a couple of doctors: (..) "Look, I feel this, and this and that doctor. (..) It's not a problem of (..), it's a disease problem, I'm sure about it. Only one [physician] is one and the other is another. [With one] the treatment would be better. Because here there is a lot of variation in the doctors here. Then, I have a consultation - I forgot to tell you that - scheduled for the 23rd, but I don't even which doctor will attend me. If it is the same person who attended me the other time, or not." (PCP 10 – BHU, male)

(4.3) Presenting prescriptions and medications

"I usually ask the patient to bring the prescription. There are patients who have a difficulty, that is, explaining what they are taking, you know. So, I can't understand through the conversation. I say "so, when you come to therapy, I would like you to bring your prescription". Don't have the recipe? Bring the medicine box, of everyone you have taking. Then I can do it.'' (PCMHP 3 – FHS, female)

''They usually show the medicine, so they take the medicine box because many do not know how to say the name of the medication, right, they do not know how to say anything, then they show the medicine and it is difficult for each other to know the name of the medication " (CHW 7 – FHS, female)

"I take the [medication] card and I take the medication with me." (PA 9 – FHS, female)

(4.4) Home visits to review prescriptions and medications

''Usually you get there [to a patient's house], they come and sometimes they even come with the little bag, you know, and then, they already show you the medication they take, there are usually all these medications they take: "I took this, took this" (CHW 7 – FHS, female)

''So they often come for consultation, we don't understand what you're talking about. It is, sometimes they go to other specialized services, which do not have a counter reference, that you know what they are doing with this patient. So we try, sometimes, to help home visits. The fact of going, being able to go to the house, say: I will go and see what, what, how, right? That he takes, for example. Sometimes he comes and says: "How are you taking the medication?"'' (RN 1 – university FHS, female)

5) Monitoring medication adherence

(5.1) Routine monitoring of health parameters

"I usually find out because they come to the appointment with uncontrolled pressure or diabetes, the medical test results are very bad. [...] Sometimes, like I said, we only know because his/her pressure is out of control, his/her exams are so bad or he/she is not feeling well, he/she has some symptoms of the disease." (PHY 6 – mixed FHS & BHU, male)

‘’Every week there is a group meeting, every Thursday we advise on hypertension and diabetes. The walking in groups take place every Monday, Wednesday and Friday. [...] Nursing staff and the doctor, the community health workers also, all help with the groups, each one speaks a little. An examination of these patients' daily hypertension is performed, every day we are followed up for fifteen days, we do glucose test every two days. If the patient is decompensated, we do it every day. And if we are totally decompensating, we do it three times a day: in the morning, before lunch and in the afternoon’’ (NA 1 – FHS, female)

(5.2) Looking out for signs of medication shortage or over-accumulation

"And, sometimes, when you prescribe medicine for the patient, he/she says: " Ah, this one you don’t have to [prescribe] because I have enough at home". Have at home? Something is wrong. Then I say: “Even though you have it, it will come out on prescription and you should take it the right way”. So, like this, it is to take them every day, it is not to accumulate them, I madke it very clear. This business of leaving a lot at home is not the [right] thing. The pharmacy delivers for a maximum of two months.” (PHY 8 – FHS, male)

“Because sometimes, the person comes to ask for a prescription ahead of time. If every 10 days she goes there and takes 30 pills, why the first day does she come here asking for another prescription? Why did the medicine run out too soon? Then I ask, "Aren't you taking one pill a day?" and then I look there, she went this month, she took thirty, it was the other month, she took thirty, it wasn't a month, hey, but why wasn't it? Either she is not taking it or she took the wrong medicine and then I ask: "How are you taking medicine?" And then you can find out through that, understand? But it’s usually asking.” (PHY 10 – FHS, male)

“Well, I already carry them [medication], where I go, they have to be with me. If I didn’t have [them with me], I'd feel ‘unwell’. Even if it's not time to take them, do you understand?” (PCP 9 – FHS, female)

(5.3) Primary care pharmacy computer records use

“The pharmacy [staff] always makes a note on the back of prescription the day it was taken and has the date of the prescription, I put the date on all of them, so if it was in August in January, it has to be taken again, January is in May again, I am doing it now it starts in September again. So, if he comes back after that, we ask him what happened. [...] Psychotropic medications are better supervised and that helps us to” (PHY 5 – FHS with TSSP, female)

“Any pharmacy within any primary care unit. If he takes, for example, an AAS [Acetylsalicylic acid] in a [primary care] unit X, I can access his history here and see that he took this medicine, the date he took it and the quantity. I can see it.” (RN/M 9 – BHU, male)

(5.4) Seeking information from family members

“Often there are people [patients] who lie, some of those are accompanied by the husband/wife, the husband/wife confirms or not. [...] He/she comes along for the consultation and tells me. The person says: “he's not taking any medicine”. I try to stay attentive for the person to pass it on [this information] to me. But is complicated.” (PHY 6 - mixed FHS & BHU, male)

“I call a spouse – “ah, for the next appointment, could you bring your accompanying person?” and try to talk to the spouse, [asking] if the patient is adhering, if is using medication, if is behaving. So there are ways for us to try to find out if this is happening.” (PHY 9 – BHU, female)

(5.5) Home visits to monitor medication adherence

“Then, in these cases [suspected non-adherence], I ask a nursing assistant, to go to the house to see if there is any medication left. Whenever I visit, I ask "how do you take the medicine?" or when it comes to the consultation “how do you take the medicine?”. [...] Then they (..) Then we can get a sense of whether it is taking right or not.” (PHY 7 – FHS, male)

“I take the prescription that the doctor gave and the medication to know if he/she [patient] is taking the right dose, make a correct calculation to see the time and day he/she is taking it, right, if he is not taking it right then I say to the patient “look, you're taking too much.” (CHW 4 – mixed FHS & BHU, female)

“I had a patient for whom we used to separate medication, so that she could take it right. Basically, she was hidding the pills, then once we went to visit and I was looking at the medication she takes, then she opened a little bottle, it was full of the medication that we had separated for her, so she didn’t take the medication even though we separated it by day, by schedule, taking the guidelines, she didn’t want it.” (CHW 5 – FHS, female)

“She [a CHW] always comes here, because she books my appointments and if she doesn’t come, she doesn’t let you down, the phone rings, she sends my neighbour. My head is forgetful, but the girls [CHWs] come here, they say: “Mrs [name], come [to PHC unit] the day” (PCP 1 - FHS, female)

“[name of a CHW] and I, we used to work together, and then she became a CHW, she started to visit the houses, and she asks everything and so she came to know more about my life, she started to be more intimate with we, she is the one who knows everything, knows about the disease, about private life, knows everything.” (PCP 3 – FHS, female)

6) Taking medication unsafely

(6.1) Not taking medication on time

"So, for example, this [medication] is to be taken after breakfast, [so patient would say] " when I don't have breakfast, I don't take medication". It happens sometimes. Then, "it's for taking after lunch, I didn't have lunch, so I didn't take it". Then I say, “even if you don't have lunch, take it at the time it would be for lunch” because [a patient] confuses things, he/she thinks it's only with breakfast, only at lunch, just at dinner ” (PHY 5 – FHS with TSSP, female)

(6.2) Using the wrong amount

‘’Sometimes you get there [patient’s home] and the medication is already running out and is not according to the schedule, sometimes it is to be taken only half a pill, but they take a whole pill, because a patient thinks he/she is a doctor and can make decisions ALONE, but in principle it’s not like that. Blood pressure, the blood pressure didn’t go down and he/she has a headache, so he/she goes there and takes two pills.” (CHW 4 – mixed FHS & BHU, female)

‘’I asked [a patient]: “Why didn't you go and get the medication? It’s already three days pass the date you were supposed to take it, and you didn't.” [...] You must be without medication”. She said: "No, I still have medication". "But how? If the pharmacy provided you with 60, yes, pills for you are taking and you should have already taken it and you didn't get it. How do you have medication?” "No, I have." "[…] On a prescription it was written that she had to take two per day, but she was taking just one.’’ (CHW 11 – FHS, female)

“Because yesterday I didn't take [psychotropic medication], today I didn't, because yesterday I had a drink, then I didn't take it (..) It's bad, right?” (PCP 19 - FHS with TSSP, female)

“It is so much medicine these days that I spent three days without taking my heart [medication], in the room I was fainting, I was very tired, very short of breath, then I realized - my medicine bag. I realized that I wasn’t taking it, my daughter even got mad: “Mother for the love of God, the most important thing”, She went running, bought it, I started taking it, it’s to take two per day. I say, one more is okay, I take it only at night, because girl, it is a lot of medicine, but then I start taking it every day, until it normalizes there, I’m fine. ” (PCP 1 - FHS, female)

(6.3) Failing to check with a doctor before stopping

"[A] lot of people think that hypertension [involves] taking medication whenever they want, when they want, you know; they stop, discontinue the treatment" (RN/M 13 – BHU, female)

“That I'm taking medicine, these medicines that you take for depression are addictive. So I start taking the medicine, [as soon as] I start to feel good, I stop. [...] they [antidepressants] to give you that thing, to give you that up in life, they throw you all the way down. Then you feel more depressed, you feel like that, a total wreck.” (PCP 8 – mixed FHS & BHU, female)

(6.4) Failing to report adverse effects

“Then I said [to a patient]: “Ah, very well. Then you felt you should stop taking what was prescribed for blood pressure because it is giving you, you think that the pressure medication is giving you dizziness? When do you get a follow-up appointment?”. ”Ah, it's next month" [patient responded]. I said: “So, you are going to do the following: you go to the [primary care] unit, at seven in the morning, you go to talk to the nurse. If it is the case, the nurse who will decide to show you to the clinician, the clinician [...] Tell the clinician what is happening

to see if it really is the pressure medicine, if it is the dizziness medication, if it is the thyroid one, what's going on." (CHW 11 – FHS, female)

"I told him already [a PHC physician], that I think Alcytam [anxiolytic drug] was already making me unwell, because it was already like this - having the opposite effect." (PCP 14 – BHU, female)

(6.5) Sharing drugs

"As I mentioned to you, there are a lot of patients that one would diagnose, start the treatment and the patient doesn't use the medication. He/she received the medication and passes the medication on to another patient who he/she thinks has the symptoms. He/she medicates other people, it has already happened, I have already witnessed several situations like this." (PHY 4 – mixed FHS & BHU, male)

''[One patient said] "Ah, but I don't know who died and left the medication''. [Another patient said] ''The neighbour stopped taking it and gave me the medicine". "(PHY 11 – BHU, female)

"I even suspect that they create a black market for medications: they simulate situations, simulate symptoms in order to obtain, for example, medications and giving to each other. I have exposed this [type of] situation a lot. People end up lacking medications, I see that it ends sooner [than expected]." (PHY 3 – FHS, male)

''In the chart we have this control: "I gave you sixty pills last week and are you coming here now to ask for more? There was no time for you to take one a day". What is the explanation for this? You're taking it wrong; you're sharing it with someone. It's impossible." (PHY 3 – FHS, male)

"Sometimes they [patients] want to keep medication. Sometimes […], I've heard of selling. There's a lot of this, you know? The guy shares… just yesterday I saw a patient, the patient was with his wife and he was here on her side. Then I said [to the wife]: "oh, I'm going to change your medication". Then he said: "wow! but now what? What am I going to use?". Because he was taking her medication. I said: "How come you are taking her medication? You have to go to a consultation to see if it is the same thing!". Sometimes it is not. We have many cases like this. Of pass- a relative passes and the rest of the family uses the same medication, so sometimes they try to get around the pharmacy to get medication, you know?" (PHY 9 – BHU, female)

7) Perceived reasons for medication non-adherence

(7.1) Difficulties with access to medication

''We have many difficulties with the patients arriving here and are hypertensive, diabetic. The patient knows he/she needs medication because cannot do without it. Arrives here on the last day the medication is finishing and wants a prescription. We have a lot of difficulty with that." (RN/M 6 – mixed FHS & BHU, female)

"Often you prescribe a medication for the patient to use, the patient goes to the pharmacy and there is no medication. On a prescription, by law, you enter the [expiry] date and it [prescription] has a validity. The patient, when he/she comes back, the prescription is no longer valid. What is he/she going to do? He/she will look for a way to get that medication when the medication arrives, and he/she will need the prescription." (PHY 3 – FHS, male)

''I think it is also an issue of access to medication, that restricts the patient care in the primary care unit, because there is no dispensation for mental health here in the [name of the FHS] unit. Antidepressants we do not have. It is centralized in the mental health pharmacy. And some non-clinical medications can be prescribed. Perhaps even can [be prescribed], but only he/she can buy - the patient. He/she will not be able to obtain it from the [public healthcare] network. It can only be done with a prescription from a neurologist or psychiatrist. Most of the patients is with a prescription of either of the two, to be able to obtain it through the [public healthcare] network. " (RN/M 1 – FHS, female)

"So, to begin with, they recover a lot from the lack of medication they are going to get, that is, I have heard in the last few days, lack of medication, here there is not, here at the pharmacy, that they go to get the medicine and there is no right [...] and they also don't have the medication, sometimes they don't have the money, they don't have the resources " (CHW 7 – FHS, female)

"I was without medication for a while, because a public [healthcare] network was lacking it" (PCP15 – BHU, female)

(7.2) Change of or multiple prescribers

"You come here to make an appointment, but you already see another doctor, that doctor gives you a medication, let's suppose; then you come here, the doctor [from here] asked for the exams and thinks it [the result] is wrong, then she gives you a medication that doesn't match that medication [prescribed by the other doctor], because that [other] takes care of his/her specialty [from secondary care], you know. Then the person [patient] deteriorates, and that person will never treat other health problems and they will not even know why, but we were told in a course we did that this counter-reference would be very important." (CHW 6 – FHS, female)

"This business of changing doctors today, changing doctors tomorrow. In the end, you are in troubles with everyone. Isn't that so? Because you are going today, then you express everything to a doctor. I even went there, talked to Dr. [name 1] and explained everything to him, okay. He gave me what [medication] was to be taken, everything. Then I went back there, it was no longer Dr. [name 1], the other doctor was there already. Now is no longer that doctor, now is a she doctor. Are you understanding how it works? So you can never deal with anyone, because then it becomes that snowball, neither one nor the other. So look, I have seen so many doctors, that I don't even know how to explain. It has been many doctors already (PCP 12 – FHS, female)

(7.3) Impact of co-existing mental health disorder

''I think the emotional illness problem makes therapy even more difficult because many times people associate their discouragement with the lack of effect, that they don't get better.'' (RN/M 3 – FHS, female)

"If there is a lot of decompensation from the depressive phase, I think they [patients] will abandon other things because they no longer care about their health, they don't care about life, so I think that in this regard, it gets in the way" (PHY 5 – FHS with TSSP, female)

''Sometimes you feel like: ''Oh my, why me!? With so many bad people [in the world], right?'' So, I had that worry. I said to meself: ''you are depressed''. My pressure doesn't go down, oh, I can't eat this, I can't eat that, because my glucose would go up. There was a time when I was like that, you know? I used to say: ''Wow, why with me?'' Damn, right? I wanted it so much, I don't even know what. Then I relaxed. I said: ''no! If it has to be, it will be. Let's take care and that's it. But I think there are people who get even worse, right? And the person can sometimes no longer succeed, that is, to get rid of this depression in relation to glucose and, and hypertension, why? Yeah, they won't be able to improve, because they focus on that [feeling], right? Seemingly it depends on this, to be able to live. You focus on that, you're not going any further, right?'' (PCP 17 – FHS, female)

(7.4) Difficulties with following instructions how to take medication

''She knew that one [medication] was for blood pressure, the other, was, it was for that health problem that the doctor there had prescribed, and the other was for the thyroid. Then she said: "Ah, I'm taking a [...]" - she even did it with her hand [finger gesture to indicate size] - "[...] I'm taking a little one, so I don't know what it is for. Then the doctor from [name of an ambulatory care clinic] prescribed me one for dizziness and I'm mixing the two. I'm not managing". (CHW 11 – FHS, female)

"I have another patient in my area, he can't read. He can read only little and his wife can't read at all and we have done several jobs with him. [...] He confuses things because he has hypertension, but [also] he has a heart problem, he has depression, and he recives treatment not only here at the [FHS] unit'' (CHW 11 – FHS, female)

''There are a lot of patients who can't even read, you know, they're illiterate, so they can't even read the prescription. [...] one of the biggest problems we face is this, of a patient taking the wrong medication, especially these diseases that sometimes take a lot of medicine, two, three for high blood pressure, two more for diabetes, plus that of dyslipidaemia, there are many medications. Patient who does not know how to read, it is difficult, right!?'' (PHY 6 – mixed FHS & BHU, male)

"It is so much medication, these days, I spent three days without taking the one for my heart, in the room I was going down, I was very tired, very short of breath, then I thought of my medication bag, I realised that I wasn't taking it [the heart medication]. My daughters even got mad: "Mother for the love of God, it's fundamental, mother!", she [one of the daughters] ran around there, bought it. I started taking it, it's to take two a day, I say, one more is okay, I take it only at night, because girl, it is a lot of medication, but ew [...] I started taking it every day, until it normalised, ew I'm already fine." (PCP 1 – FHS, female)

‘’I take diabetes medicine, blood pressure, and there are these medicines here that I don't know how to take, only for that I stopped. I can’t manage, right. I can’t manage’’. (PCP 5 – mixed FHS & BHU, female)

(7.5) Beliefs about consequences of a medication use

‘’Oftentimes it is more the matter of behavior, that they do not adhere to treatment, do not accept the imposed medication, not due to difficulty in accessibility. Ah, it is the matter of adherence, to think that the medication has no effect, that it is doping him/her, that he/she does not need it.’’ (RN/M 3 – FHS, female)

‘’They [patients] often tell, you know: "Oh, I didn't use it, I used it once it gave me stomach pain, it gave me diarrhoea, I didn't want to". Something like that, understand? And there are some forms, either by their stamp or by, or checking at the pharmacy if they left, or the patient even saying that he didn't want to use it. He used it and it didn’t work or “Oh, I thought about it, but the neighbour told me about this medicine, I got afraid and don’t want to”. (PHY 11 – BHU, female)

“There are a lot of patients who don’t take it, who think the medication isn’t helping, that it’s giving side effects” (PHY 3 – FHS, male)

‘’She [a primary care physisican] medicated me, you know, but I told her straight way: “I’ll be sincere saying, that I am a semi-complicated patient.” I'm taking medicine, these medicines that you take for depression that are addictive. So I start taking that medicine, as soon as I start feeling good I stop. Huh. She even fights with me: “This is not how you do it, you have to follow the treatment!”. I say: “doctor, but this thing is addictive!” [...] they [antidepressants] to give you that thing, to give you that up in life, they throw you all the way down. Then you feel more depressed, you feel like that, a total wreck.” (PCP 8 – mixed FHS & BHU, female)

“When they told me it was fibromyalgia, that they couldn't do anything there, that I was supposed to come back [follow-up], continue with the post [attending the FHS unit], I went! Then they passed Amytril [Amitriptilyne; antidepressant, anxiolytic, anti-pain drug] to take, right? [Amitriptyline], and that's it. Then I went to the appointments, I spoke, but ... Nobody did anything! So, in terms of what I’m talking about, of doing nothing... I spoke about pains, everything. There have been people I had to listen to tellign me to learn to live with pain, right?! So I gave up for good. So I’m almost don’t, don’t chase after. I will not lie [to you], I will tell the truth! (PCP 12 - FHS, female)

(7.6) Religious beliefs interfering with medication use

‘’There are patients who, depending on their religion, they are, they check with the major representative of their religion whether to take such medication or not. I even had a case of a patient who interrupted the medication because the major representative of the religion said “look, there is no need because you will be cured by, by God, you will be cured” - usually they speak - “by a superior being”, “ God ”." (PCMHP 1 – FHS with TSSP, female)

‘’There is a case of a guy there, who became a Jehovah’s Witness, and that he abandoned [the treatment because] Jesus was going to save him’’ (PCMHP 4 – FHS, female)

“That faith. I believe in God. God will heal me. I'm sure I'm going to get out of this here. I ask God: “Lord, help me, take this medicine [a lot of medicines] from me”.” (PCP 9 – FHS, female)

(7.7) Difficulties with incorporating medication in daily routine/lifestyle

"The medications themselves, often it’s a matter of having to take them at the right time, sometimes they don’t [take them] because they do not have the network and the person has to have a financial expense that they did not have before, quality of life, that some habits will have to be changed, some [daily] routines will have to be changed, the fact of having to come to [see] the doctor more often. " (RN/M 3 – FHS, female)

“Yesterday I didn't take [my psychotropic medication], today I didn't, because yesterday I drank [alcohol], so I didn't take it (..) It's bad, right?” (PCP 19 – FHS with TSSP, female)

8) Most challenging health behavior change goals

(8.1) Lifestyle changes

(8.1.1) Lack of commitment to making lifestyle changes

"[Some patients] do not take care of themselves anymore, they will not try to do the prevention, they will take care of themselves by taking the medication, some even change habits, start doing physical activity, start to change their diet, others do not, others think that no matter how much we advise that with the medication only, he/she will be fine. " (RN/M 5 – FHS, female)

‘’They [patients] prefer to take medicine rather than to remove salt [from their diet], or to do physical activity, you know? And diabetes the same thing. What can we do?’’ (RN/M 14 – BHU, female)

"[A doctor wanted to refer me to] a nutritionist, for me to speak with her. I said: “don't give me a doctor because I don't want to”. He asked:" You don't want to lose weight? I said: “no, doctor, I don't want to go." (PCP 6 – FHS, female)

(8.1.2) Lack of understanding of the importance of lifestyle change

“Some elderly people, they have a culture very (..) it is their culture, this is the education they have had, for a long time, that doesn’t allow for many things. They think this way: that they are eating something that will make them unwell, we know that this is not doing them good, that it will be bad, but they ate it their whole life; it is not like it will change now, that it will improve for a reason. He/she doesn't accept it, there are people who don't accept it. We respect it. At that moment, we respect it, but keep trying to show that that is not right, that it is wrong, that it has to change.” (CHW 9 – FHS, female)

‘’I think they only know about pharmacological treatment. They are a lot like that, it's medication, medication [...] When you talk about the importance of physical activity, many think it's a lie. Many think that it’s enough to take the medication to be allowed to eat whatever they want. It takes a long time to wake up to the reality of having to go on a diet, it takes a long time, it is not an easy thing.’’ (RN/M 3 – FHS, female)

“Then the doctor started explaining what the diabetic was like, he said “The diabetic is like that, we have it in our body behind our large intestine, it’s called the pancreas, so everything you eat, so your pancreas is no longer working normal, like that person who doesn’t have diabetes. The one who doesn’t have diabetes, he eats and the pancreas immediately throws insulin on what that person ates, and it destroys it, and yours is no longer working well, it’s not okay, so it doesn’t throw insulin, where what goes in the blood and in the blood it turns into sugar, because if you eat bread, something with fat, sugar, everything you eat turns into blood sugar, and then it’s bad circulation.” Well he explained everything, and then the receptionist came, there was a big screen, like this, a big paper on the wall, and she wrote, she wrote in block letters, what I could eat and what I couldn't eat, she wrote on the wall, and at that point, I ended up with doing that thing. I didn't eat ice cream anymore, fizzy drinks, I never had fizzy drink since. I’ve never ate chocolate anymore, so I drink coffee with milk and sweetener, right.” (PCP 7 – FHS, male)

(8.1.3) Difficulties with changing and creating new habits

"They [patients with difficulties with changing eating habits] are all elderly people and they have a great difficulty with nutritional re-education, very great because the eating habits come from the father, from the mother, so [it’s] difficult, now to enter with fruits and vegetables, educate the right amount to eat, they don't know that at all." (CHW 6 – FHS, female)

"What I observe, is that people adapt to the disease, what happens [is that] the patient thinks that hypertension and diabetes is an organ that is a part of them and in reality it is not, these are habits that must be changed, but for an elderly person changing 30 to 40 years old habits is almost impossible, you have to go lightly, work month by month then he/she starts to understand. When he/she is at the moment when starting to feel unwell, then you explain that he/she has to look for health care in the primary care service network. [...] The Brazilian has very old customs and habits, has traditions that make this society complicated.” (CHW 4 – mixed FHS & BHU, female)

“She [a doctor] wants me to walk, but I can't walk. I have a walker and there are days when I have to walk inside the house woth a walker. I can't even walk alone. I'm afraid of falling, because I'm fat. So I am very afraid of falling and breaking something, and then having to stay in bed, which is my dread, my daughters will not be able to take care of me. Only how well I know how hard it is." (PCP 1 – FHS, female)

“I felt good, because he gave me a correct explanation, I was in doubt and he answered my questions. I remembered his guidance. I said: “I'm going to start walking to see if I get better”. I said to him: “Doctor, would a treadmill do the job?” Then he said to me: “No, only on a rainy day, because when it's not raining, you go for a walk, you meet people and then you say ‘hi how are you’, you meet another person and it’s better this way.” He said that what the walk helps with, also helps your psychological [well-being].” (PCP 3 – FHS, female)

(8.1.4) Emotional dimensions of unhealthy habits

"When one has the two [diabetes and hypertension] diseases, then it gets a little complicated, because I have to make him/her understand, that both sugar and salt [are harmful] until they manage to reduce, but the sugar gives him a certain satisfaction, causes a kind of (..) emotional relief and is complicated to give up on. " (CHW 4 – mixed FHS & BHU, female)

‘’I think this issue of diabetics I see this very clearly. The person is used to eating candy for many years, you know? And then he/she can't. Sometimes it happens. They get sort of ‘depressed’.’’ (RN/M 1 – FHS, female)

"I think that many people get really depressed, many get [depressed] and I think the fact that they can't follow a diet, they can't, is because it’s like you restrict the best thing these people have which is to eat, you ever thought about it? " (RN/M 14 – BHU, female)

"I've always been chubby, since I was a kid, and at that time, no one cared about it […] None of that: “not like that, I do not eat that, no.” “It's chubby, it's beautiful, it's wonderful, it's bla bla bla.” […] So you went to grandma's house, there was a cake, there was canned condensed milk (..) So you did not go against it. Maybe if in the past they had already had this concern, today we would not be suffering what we suffer." (PCP 17 – FHS, female)

(8.1.5) Social influences on lifestyle choices

‘’There is an association between food and love, it exists in our culture, in our way. I have, for example, patients who say: "the only way she gives us love is that cake, that food she makes". There is a cultural association that food is love. And sugar is also associated with that.’’ (MMHS 3 - female)

‘’She [a patient] knows that she has to decrease her salt intake. However, the rest of the family, the husband and children, do not understand. They complain about the food being made and do not support it. In the case of diabetics, many times, others [other family members] like sweets and end up bringing them home, which creates an illness.’’ (NA 2 – mixed FHS & BHU, female)

“Even during the weekend, I go down to [a name of a place] to go hiking, but it's not the same. Is very bad. Now, with the group, it is totally different. [..] getting out of that little world I was in and doing the group walk is how my depression improved.” (PCP 8 – mixed FHS & BHU, female)

(8.1.6) Limiting environmental context and resources

‘’They say to me: “Ah, doctor, I’m a little upset”, they [patients] use that term - “I’m a little upset”. Why [are you upset]? “Because I can’t eat such a thing, I don’t have the financial conditions to buy a healthier food, a food that will be more, a food that (..) Sometimes I need a specific food [special diet] and I will not have financial

“The poorest population does not have access to a diverse diet so I can’t, I can’t even try to demand a diet from that [kind of] patients, that is, fruits and vegetables, since he/she doesn’t have the money to buy it. He/she has money for the basics. [I tell the patient:] “Oh, I wish you were exercising”, but he/she works all day. So sometimes he doesn't have time to exercise more, so many [patients] come and say: “oh, but I already work all day, I want to go home, I'm tired”.’’ (PHY 9 – BHU, female)

"My granddaughter arrives and asks for things: "Grandma you don’t have anything [to eat] here?". [I respond]: "No, love"; “[Grandma, but] I'm hungry". So I get already nervous, because I do not have it, you know? This gives me, gives me depression, lack of things even, sometimes I feel like eating something but do not have money" (PCP 15 – BHU, female)

(8.2) Depression and anxiety drugs abuse and misuse

(8.2.1) Incorrect use of antidepressants and anxiolytics

‘’A patient also, sort of, doesn’t understand the treatment [for depression]. There are patients who think that the medication for depression, for psychiatry is ‘just taking medication’, and also taking it when you are not feeling well. [...] Then: “Have you taken your medication?”, “Ah, the day I see that I'm not well, I take it”. I mean, there is no point in taking a fluoxetine one day.” (PHY 8 – FHS, male)

“That I'm taking medicine, these medicines that you take for depression are addictive. So I start taking the medicine, [as soon as] I start to feel good, I stop. [...] they [antidepressants] to give you that thing, to give you

that up in life, they throw you all the way down. Then you feel more depressed, you feel like that, a total wreck." (PCP 8 – mixed FHS & BHU, female)

"The drinking? Phew (..) Hasn't decreased a bit, gosh not at all (..) It is difficult, you know? I take [sertraline; antidepressant]. I mean, I take it when I don't drink, right? […] When I don't drink, I take it, but now when I notice that I'm drinking again, then I don't take it anymore. I do not." (PCP 19 - FHS with TSSP, female)

(8.2.2) Widespread use of benzodiazepines to manage sleep problems

''[A] lot of people here use sleeping pills. Many people. It is what most caught my attention when I arrived at the unit. Everyone takes benzodiazepines to sleep.'' (PHY 9 – BHU, female)

''Everyone here takes medicine for depression. It's easy, access is easy. You come and say: "Oh, doctor, I can't sleep", and "man" [here it is], diazepam. It is an antidepressant. Most take diazepam here, half a pill, one at night. In the region, it is one of the cities that most provides controlled medication.'' (CHW 8 – FHS with TSSP, male)

"No, this [an antidepressant] not, I just take one diazepam from there [a MHCC], right? From there [I take] only diazepam. Only, now the stronger ones do not, diazepam helps to sleep, but it's not helping me, I am still not able to sleep. I didn't sleep this night. […] There are people there who take those strong medicines that turn them into a parasite, it is like with (..),I don't know, like they have medicine there that makes you crazy, you are not crazy, but that leaves people walking around. No, I already took these. I already did. But I didn't take it afterwards, I got bad. I told you I was unable to walk at all, I had to go to the emergency department (..) in an ambulance." (PCP 19 - FHS with TSSP, female)

(8.2.3) Antidepressants and anxiolytics dependency

''The abusive, excessive use of benzodiazepines, something the patient doesn't accept, [patient says] "No, I want, I want, I want!", You know? It is the dependence on benzodiazepine that is what scares me the most here, in fact, that clonazepam, prezolan, diazepam, so it is an addiction that you have. Who wants it anyway and you try to say no and they, you know, get aggressive even. That's what bothers me most, the benzodiazepine addiction. (PHY 5 – FHS with TSSP, female)

"They are all like that, patients with depression they are very dependent on medication, so they can't live without medication. So much so that the prescription is expiring, they are already deranged, they [feel desperate] need to schedule [an appointment] to renew the prescription. The concern is not to consult with a psychiatrist or psychologist, it is the medication, they cannot be without it.'' (CHW 2 – FHS, female)

"I couldn't sleep without the medicine [benzodiazepines], do you understand? Wow, I didn't sleep all night, so I'm getting addicted on it, so I think it's good to see if I can get it off." (PCP 15 – BHU, female)

"I said these days, I told her [a FHS physician] that I took two pills of lorazepam to get to sleep, she said nothing, she did not answer anything." (PCP 2 – FHS, femeale)

"I said that I (..) felt dispirited, then he [the BHU physician] gave me this medicine for me to take to sleep. To take at night. So I started taking it. (..) But it started to make me very sleepy during the day, so I talked to this doctor, all that. I said: "It's like this, like this, like this", but at the time the doctor has already (..) stopped prescribing it. Then she prescribed this [new] one for me to take in the morning, (..) I don't even remember his name, it is a medicine, I also think, controlled." (PCP 10 - BHU, male)

(8.2.4) Alleged negative social influences on the use of depression and anxiety medication

"[patients saying] ''Oh, I thought about it [an antidepressant], but the neighbour told [me] about this medication, I got afraid and don't want to". (PHY 11 – BHU, female)

"For depression, a patient wants to know about [a specific] medication, because it's like this: he took someone's medication, he/she had that thing where can't sleep and there is always someone who gave a medication at home, who gave it to that person. "Ah, that medication was very good for me, doctor!" (PHY 8 – FHS, male)

"[A] patient [feel like] has to take the medication that the neighbour takes, that wants to take the same, many take it, and it happens that he/she takes one [antidepressant] without going to the doctor and thinks that this will treat him/her''. (PHY 3 – FHS, female)

"I went to the MHCC because I wanted to, you know? Because my son was going there (..) Because he had problems too, right? And then I went there. Because he was in prison, then the judge asked as a part of his

sentence to go to the centre MHCC, right? Then I went, I enjoyed it and we stayed there together." (PCP 19 - FHS with TSSP, female)

"As I told him [a PHC physician], that I think Alcytam [anxiolytic drug] was already making me unwell, because it was already like this - having the opposite effect." (PCP 14 – BHU, female)

"She [a primary care physician] medicated me, you know, but I told her straight way: "I'll be sincere saying, that I am a semi-complicated patient." I'm taking medicine, these medicines that you take for depression that are addictive. So I start taking that medicine, as soon as I start feeling good I stop. Huh. She even fights with me: "This is not how you do it, you have to follow the treatment!". I say: "doctor, but this thing is addictive!". (PCP 8 – mixed FHS & BHU, female)

"Out of the blue I felt my heart racing, so I saw that it was indeed anxiety, and so from then on I started to take a tranquilizer, I needed to take it. The thing is that I didn't take it correctly, you know? It was due to a ... (..) I think it was a lot due to the suffering, and I have within me, in my head, that there is something here inside my heart that hurts me. (..) So I think that's where I started to realize, you know? But that was about thirty years ago, that's where it came from, and my anxiety has been worsening since, you know? […] And there was a time when I got really doped. The psychologist yes, I miss having one. But, not (..) not a psychiatrist, no. Because thanks God I stopped taking that medicine. I manged to do it. God took me out of it. I stopped little by little. (..) I said: "God will get me out of this, this thing." Why to stay in that world of medicine, right? This is bad for us. (..) Then thanks to God, Tryptanol [Amitrytpynilne; trycyclic antidepressant] and diazepam [benzodiazepine; anxiolytic drug], I stopped a long time ago." (PCP 12 – FHS, female)

(8.3) Insulin injections

(8.3.1) Reluctance to use insulin therapy

"When a diabetic gets to the point of having to take insulin, <u>holy mother</u>, they are very difficult, very resistant, a very resistant person." (CHW 7 – FHS, female)

''How difficult it is to introduce insulin to the patient, because they want to continue doing oral therapy but don't want to [insulin], because until they are using insulin, they don't feel diabetic, right?'' (RN/M 14 – BHU, female)

''[patients say] "I'm going to have to use insulin. Wow, my life is over. I'll be using this needle for the rest of my life!". So they already look at it in such a pessimistic way. And then that person will have a problem, right? The patient does not accept the use of insulin, does not accept the disease.'' (PHY 11 – BHU, female)

"Here in the [BHU] unit [I discovered I had diabetes]. (..) Then, they gave me the medication, right? Including a (..), there was a she doctor, after Dr. [name] left. At the second appointment she wanted me to (..) my blood pressure, my diabetes was high, and she wanted me to take insulin. So I said: "For the love of God!" That I already had one case in the family, right. That is my son, he takes insulin. (..) So I saw his suffering, those things there, so I asked her for medicine (..) without insulin. (PCP 10 – BHU, male)

"In a sense it gets a little uncomfortable, right? Uncomfortable, you know, to apply, to prick my finger, to apply to the belly." (PCP 15 - BHU, female)

(8.3.2) Efforts to overcome patients' resistance to insulin therapy

"When a patient starts any type of treatment, especially insulin therapy, then we call [the patient], show what will happen, how it works, so like, do all this guidance and when a patient gets on insulin, the pharmacist also does this part, because as she distributes the glucometer, she also does this orientation part. So, then, in addition to providing guidance here, the doctor does it, the nurse does it and the pharmacist also ends up participating in these types of guidance, understand?'' (RN/M 14 - BHU, female)

"Some cases, more complicated ones, that we are unable to monitor, when a patient uses insulin, for example, at home and nobody wants to apply it and he/she [the patient] does not know how to apply it alone, they [PHC staff] give the medication at the clinic [the mixed FHS-BHU unit]. Sometimes, there have been some patients that I asked to take medication at the clinic. They go there in the morning and at night. It is not ideal, you know, the right thing is to try to make them take it at their house, but when there is no way, it is complicating his illness and he is not taking the medication properly, we do it that way. Then the nursing staff do their medication." (PHY 6 – mixed FHS & BHU, male)

(8.3.3) Endocrinologist referrals when efforts to monitor insulin therapy failed

"The same thing, diabetes, many times we try to keep them at the unit as much as possible progressing with the oral medication, then the insulins come in, and then we have to refer to anendocrinologist.'' (PHY 9 – BHU, female)

“I'm going to have to use insulin. Wow, my life is over. I'll be taking this needle [to my body] for the rest of my life!”. They [patients] already look at it in such a pessimistic way. And then that person will have a problem, right? The patient does not accept the use of insulin, does not accept the disease,’’ (PHY 11 – BHU, female)

9) Main motives for initiation or maintenance of treatment

(9.1) Motivation to maintain treatment

‘’The doctor told me, my doctor Dr [name] said to me: “People who do constant walking don’t even have to take medicine, right? [...] I said to myself: “I'm going to take an hour and go for a walk, even if it's only in my neighourhood, right? It was good, I felt really good. I felt really good, so I carry on with it.’’ (PCP 16 – FHS, female)

“[A doctor said:]’’We are beginning to understand that you really have a serious health problem, so you have to take action’’. And this is what I did [...] I became aware. So, I started the treatment. [...] You are getting older, more experienced. You end up becoming aware of what really happens and that you have other people who depend on you and you depend on you. You have to take care." (PCP 17 – FHS, female)

(9.2) Behavioral regulation of treatment adherence

‘’Who taught me [to apply insulin]? It was the pharmacists inside the [BHU] unit, where I went to get it [insuline], right? I stopped by to get it here [the pharamacy], then they explained how I did it, right, the application. In the sense it gets a little uncomfortable, right? Uncomfortable, you know, to apply, to prick my finger, to apply to the belly, but over time you overcome it.‘’ (PCP 15 – BHU, female)

‘’Today I take Glicazida [medication name] in the morning, one and a half pills. I take, the Clifase [medication name] and the SSRI [selective serotonin reuptake inhibitor], it is two after lunch, two after dinner. I policed myself, regarding food. [...] But it is so difficult today, right? With so much good stuff that exists today, but you have to police yourself. And I ‘closed my mouth’ [resisted], as they say, I learned new habits, I managed to lose twelve kilos, right?’’ (PCP 17 – FHS, female)

10) Methods deployed to improve medication adherence

(10.1) Social network involvement

‘’Then I ask: “Please, could a companion come [with you next time]?”. Even if the patient is not elderly. Bring your friend, your mother, your partner, your neighbour. Someone who will be with him/her and who will understand and who will get us to work with him.’’ (PHY 9 – BHU, female)

“I talk to those responsible [for the patient], because the elderly patients by law cannot be alone, and I inform the people who take care of them.” (CHW 4 – mixed FHS & BHU, female)

"There are patients whose daughter sometimes comes, a father comes, if there is someone who cares but doesn’t separate [medication], then the nurse separates. [But] usually we look for someone in the family who can do this for the person, a father, a brother, a neighbour, a son." (CHW 5 – FHS, female)

‘’He [patient’s husband] said“ It is not like that, you cannot do this, you cannot throw yourself in bed because you have diabetes, diabetes will not kill you, what will kill you is you becoming despondent, because you are too discouraged, it’s not like that.” He said: "Go out and talk to the doctor, she will explain what you have to do", that's where I went at that time and started to treat" (PCP 2 – FHS, female)

“Even at the weekend, I go down the highway to go walking, but it's not the same thing. It’s very bad. Now, with the group, it is totally different. [...] leaving that little world I was in and doing the group walk is my depression has improved.” (PCP 8 – mixed FHS & BHU, female)

“Then, I went after my niece because my children don't visit here, they work, and they don't allow me to work. Then she [the niece] said: "No, aunt, you can let me come and give the medication to Ma'am the right way". (PCP 9 – FHS, female)

“Then Brother [name, the deacon] came here. [I said:] ‘’Brother [name], I have this prescription, the first one, but I don't have the money to buy, brother. [...] [He] bought the medicine” (PCP 7 - FHS, male)

(10.2) Extra measures for patients in need

“If we are going to pay special attention to those [aspects], yes, in this aspect, yes, so that they do not lack medication for them to take it correctly, the medication for the elderly who have difficulty, we make a table fold in the closet, or roll the medication, separate, others do not read, so we do our best to be able to help and bring the family together, try to bring the family to them they helped, so they are not dependent on us. […] Only if it is an extremely case, there is no family, there is no one, it does not work, he is elderly, then we keep giving special attention, but if the person has a family we do a search in the family and pass everything for the family to develop.“(CHW 8 – FHS with TSSP, male)

''It depends on the patient, same thing if the patient is bedridden, domiciled and such, can't move, and we can't get anyone to do that for him, like a son, a neighbour and such, then we do and if the patient adheres to the treatment, the nurse is available to separate the medications. So when we separate, we keep the prescription - we the nurse - she takes the drugs that the person needs, she separates and we take them, usually the community worker of the patient in question takes the medicine to the patient, already separated, in these cases. There is a patient who has difficulty just to separate the medication, but he can get it, the nurse separates it, he searches.’’ (CHW 5 – FHS, female)

“It should be like this: make appointments on the day so that you can better attend the patient, right? (..) But it is not like that. Booking a lot of people who get little time for attendance. (..) And you end up leaving with the prescription, knowing that you will have to continue taking medicine and trust in God, Hhhh, right? (..) There is no other way, no.! (PCP 4 – FHS, female)

“I think they [a BHU team] should be more on top of it, right, because every six months, what if something suddenly happens, right? You should give more space [for check-ups], right? Imagine you take insulin for six months and insulin is not working, and you need to you wait to see a doctor to tell her, right? Yeah, the time needs to be shorter than that, right? (PCP 15 - BHU, female)

”If I had relapses or if I was not well, for me to get in touch with her [a physician] for her to attend me me she gave me her private mobile phone number so I could call or text her. There was one time when I contacted her, to be able to be attended, as I got very upset about my situation at the time. I sent it [a message] through WhatsApp. I remember it was just before the weekend, around Thursday, Friday, she attended me on the following Wednesday. She answered the other day with a date of an appointment booked. I felt very well attended, I was very satisfied with her service. I didn't ask for her private phone number, she gave it to me. And I am very grateful to her, even today, for this attention she gave me as a patient, to pass her private phone.” (PCP 13 – mixed FHS & BHU, male)

“I have, I have to explain. I explain. I explain, I think have already explained for a couple of doctors: (..) “Look, I feel this, and this and that doctor. (..) It’s not a problem of, it’s a disease problem, I'm sure about it. Only one [physician] is one and the other is another. [With one] the treatment would be better. Because here there is a lot of variation in the doctors here. Then, I have a consultation - I forgot to tell you that - scheduled for the 23rd, but I don't even which doctor will attend me. If it is the same person who attended me the other time, or not.” (PCP 10 – BHU, male)

“No, the care provided by the doctors here (..) they, they themselves are not bad. I understand (..) the situation. They come, they ask, they can’t do any more because, I think there are no conditiona to domore, right? But they are very attentive. (..) You came through the door, in the consultion room, and it's another world. For the sake of compaling, the only only thing I could complain about is the period of attendance. It’s very long, I already had here a return - another appointment [follow-up] – a return, I think is what a doctor would define it. They, there, in the front [at the reception], they booked at four, five, six months. I've even had one year [follow-up appointment] here. So, in the case of diabetes, because it is a diabetes and I already had a heart problem, like high blood pressure, everything comes in one. I had a problem of diabetes, stroke, [myocardial] infarction. So I think I should have a closer care, right? Because they say it's a ‘deceiving’ disease. You don't know what can do to you tomorrow.” (PCP 10 – BHU, male)

(10.3) Strategies to assist with organization of medication

“So [I will know, if he is taking the medication], because we also do and organize it. There are patients who use a lot of medicines, so we organise them by bottle. Then we put a glove, sun, soup plate, to indicate, right. The hours and we paint the caps. Let's suppose, the little red is a pressure medicine; the blue whiting is diuretic

or for the heart, you know. We separate by colour and time, like that, with the drawings.” (CHW 8 – FHS with TSSP, male)

‘’We have a case of three patients, father and son (..) father, mother and son, all three [of them] take medication. The father is diabetic, the mother is hypertensive and diabetic, and the son has a psychiatric problem. These three patients are cared for differently because there is no one to take care of them, they have a family, but the family does not accept it like that, they do not want to be involved in this. The health worker in their area takes the medication in the morning, leaves it there. Oh, place the mother's [pillbox] in the bedroom, the father in the kitchen and the son places in the living room. This is more [enhanced] care - then nursing technician, the health worker goes there and takes it [the medication], she takes in the afternoon and in the morning. She goes twice a day to their home. EVERY day. Even on Saturday and Sunday she leaves the pillboxes. Three pillboxes in the kitchen, three in the living room […]“ (NA 5 – FHS, female)

(10.4) Enabling access to prescription medication

‘’So, sometimes, we end up overloading the doctor because you won’t leave the patient without medication. You overload the doctor and that nervousness that: "Oh, I will make it, I will not make it, right?". And for us, as a nurse, that anxiety that we have to get the medicine for the patient. Because how am I going to go on a holiday, or [enjoy] Saturday and Sunday knowing that I left that lady [patient] without medication. So, everything is like that. It could be easier, you know?” (RN/M 6 – mixed FHS & BHU, female)

“Convincing him/her [a patient] to use the medication, he/she then go on an uses the medication, but then we have the limitation of the medication available on the [healthcare] network. A patient here has a [certain] socioeconomic level, so we are unable to use the medications that are, let's say, the most recommended for that disease or that are (..) that have the best result, that studies show that had the best results, that patient does not need taking as many times, the patient can control something [disorder] so that it gets closer to the normal. There's no way, we work with medication that is given on the [healthcare] network. If he/she [patient] often doesn't find it, he doesn't use it so it's useless, I have to know what I'm going to prescribe.” (PHY 9 – BHU, female)

“It depends, sometimes we reach out to social services, because we have had cases like this [without acces to medication] most of the times when they run out of the medication they is unavailable through the [health system] network, they buy it themselves, it’s like two hundred, three hundred reais per medication, it’s a lot.” (CHW 5 – FHS, female)

“We phone around [PHC] units, when the person (..) when the patient doesn’t have his/her own prescription. Because in fact, we have to get that medication during the visit, but as the patient doesn’t have the prescription, because it’s often retained at the place where the medication was bought, we look around the other units and it’s when the other nurses provide us with them [prescriptions].” (CHW 1 – FHS, female)

"So, medicines from the Popular Pharmacy [pharmaceutical assistance program] help. Wow, I think it helped a lot in controlling pressure, diabetes and asthma, this is very important. Very much." (PHY 6 – mixed FHS & BHU, male)
